# Osteosarcoma-targeted Cu and Ce based oxide nanoplatform for NIR II fluorescence/magnetic resonance dual-mode imaging and ros cascade amplification along with immunotherapy

**DOI:** 10.1186/s12951-024-02400-z

**Published:** 2024-04-04

**Authors:** Mo Cheng, Qingjie Kong, Qing Tian, Weiluo Cai, Chunmeng Wang, Minjia Yuan, Wenxing Wang, Peiyuan Wang, Wangjun Yan

**Affiliations:** 1 Department of Musculoskeletal Surgery of Shanghai Cancer Center, Fudan University, Shanghai, 200032 P. R. China; 2 Key Laboratory of Design and Assembly of Functional Nanostructures, Fujian Institute of Research on the Structure of Matter, Chinese Academy of Sciences, Fuzhou, 350002 P. R. China; 3 Department of Chemistry, iChEM (Collaborative Innovation Center of Chemistry for Energy Materials), Shanghai Key Laboratory of Molecular Catalysis and Innovative Materials, State Key Laboratory of Molecular Engineering of Polymers, Laboratory of Advanced Materials, Fudan University, Shanghai, 200433 P. R. China; 4 Department of Orthopedics, Shanghai General Hospital, Shanghai Jiao Tong University School of Medicine, Shanghai, 200080 P. R. China; 5 Department of Neurology, Qingpu Branch of Zhongshan Hospital, Fudan University, Shanghai, 201799 P. R. China; 6 Shanghai Qiran Biotechnology Co., Ltd, Shanghai, 201702 China

## Abstract

**Background:**

As the lethal bone tumor, osteosarcoma often frequently occurs in children and adolescents with locally destructive and high metastasis. Distinctive kinds of nanoplatform with high therapeutical effect and precise diagnosis for osteosarcoma are urgently required. Multimodal optical imaging and programmed treatment, including synergistic photothermal-chemodynamic therapy (PTT-CDT) elicits immunogenetic cell death (ICD) is a promising strategy that possesses high bio-imaging sensitivity for accurate osteosarcoma delineating as well as appreciable therapeutic efficacy with ignorable side-effects.

**Methods and results:**

In this study, mesoporous Cu and Ce based oxide nanoplatform with Arg-Gly-Asp (RGD) anchoring is designed and successfully constructed. After loading with indocyanine green, this nanoplatform can be utilized for precisely targeting and efficaciously ablating against osteosarcoma *via* PTT boosted CDT and the closely following ICD stimulation both in vitro and in vivo. Besides, it provides off-peak fluorescence bio-imaging in the second window of near-infrared region (NIR II, 1000-1700 nm) and Magnetic resonance signal, serves as the dual-mode contrast agents for osteosarcoma tissue discrimination.

**Conclusion:**

Tumor targeted Cu&Ce based mesoporous nanoplatform permits efficient osteosarcoma suppression and dual-mode bio-imaging that opens new possibility for effectively diagnosing and inhibiting the clinical malignant osteosarcoma.

**Supplementary Information:**

The online version contains supplementary material available at 10.1186/s12951-024-02400-z.

## Introduction

As one of the most common malignant and aggressive orthotopic bone cancers, osteosarcoma is locally destructive with highly metastatic and affects both children and adolescents, accounting for approximately 50% five-year survival-rate [1–3]. Recently, combination regimens of chemotherapy and surgical resection still the two remain major therapeutic schedules for osteosarcoma patients [4–7]. Nevertheless, the inherent features of osteosarcoma like invasive progression, tightly adhered surrounding normal tissues induce it difficult to thoroughly ablate the cancerous cells during surgical removal and clinical in situ recurrence induce even worse overall survival in osteosarcoma patients [8–10]. Concurrently, the frequently-used chemotherapeutic agents, like doxorubicin, cisplatin or methotrexate that synergistically exert cancerous cell killing efficacy [11, 12], while they still have deficiencies like low selectivity and sensitivity toward osteosarcoma cells as well as the systemic toxicity of the high doses, for instance, emesis cardiomyopathy, alopecia [13]. Simultaneously, the most advanced diagnostic techniques with higher sensitivity to osteosarcoma are badly in need of establishing for early delineating and therapeutic effect monitoring, especially eradiating against minimal lesions [14–16]. Accordingly, it urgently continues to exploit fascinating options equipping with robust sensitivity for integrating the diagnosis and improving the cure rate of osteosarcoma.

As the reactive oxygen species (ROS)-related tumor dynamic therapy and new therapeutical concept, chemodynamic therapy (CDT) acts as drug-free modality and can efficaciously utilizes the high overproduced H_2_O_2_ for strong oxidative hydroxyl radical (•OH) generation in the microenvironment of tumor tissue *via* Fenton/Fenton-like reaction [17, 18]. Nevertheless, Fe^2+^-based Fenton reaction substantially requires a highly acidic microenvironment (∼ pH = 3–4), furthermore, the extremely low efficiency of this reaction rate (∼ 63 M^− 1^s^− 1^) even only occurs under the optimal pH level [19–21]. To date, transition-metal ions, like Co^2+^, Cu^2+^ and Mn^2+^ have been utilized in CDT on account of their outstanding Fenton-like catalytic activity [22–24]. Furthermore, the valence transition of Cu^2+^ to Cu^+^ is capable of being achieved with the consumption of GSH [25]. Particularly, Cu^+^-based reaction rate reaches more than 160-fold (∼ 10,000 M^− 1^s^− 1^) higher than that of Fe^2+^, in the bargain, the depletion of elevated GSH level could also greatly attenuate the scavenging effect in tumor cells that fundamentally strength the CDT efficacy [26, 27]. In particular, Cu-based nanomedicine with exceptional biocompatibility has played an increasingly significant role in multifunctional nanoplatform for tumor diagnosis, for instance, it can act as an effective contrast agent with amplified signal for magnetic resonance imaging (MRI) [28, 29]. As various CDT nanoagents have been earnestly explored, among the lanthanide elements, cerium based nanotherapeutics have received widespread concern on account of their exceeding redox feature and capability to realize reversible transformation between Ce^4+^ and Ce^3+^ for converting H_2_O_2_ to •OH [30–33]. In concordance with Cu^2+^, Ce^4+^ has also contributed to accelerate GSH consumption through redox reaction, therefore attenuating its •OH scavenging efficiency. Besides, external energy fields, especially like heat and ultrasound are capable of serving as adjunctive strategy to augment the Fenton/Fenton like reaction for ROS boosting [34–38]. As a clinical approved fluorescent probe and photothermal agent, indocyanine green (ICG) is extensively engineered in photothermal therapy (PTT) [39–41] and fluorescent bio-imaging, especially in the second window of near infrared region (NIR II, 1000–1700 nm) [41, 42]. However, the currently available Cu&Ce alloyed CDT nanoagent equips tumor microenvironment stimuli and with ICG delivery for integrating synergistic treatment and diagnosis are rarely reported.

**Scheme 1:**
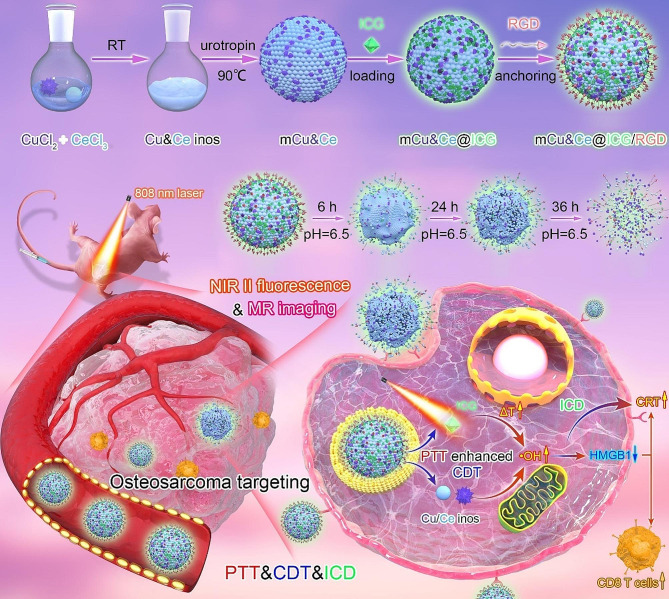
Diagrammatic illustration of construction process of osteosarcoma targeted mCu&Ce@ICG/RGD for NIR II fluorescent/MR bio-imaging and synergistic tumor suppression by PTT-CDT-ICD.

Notably, immunogenic cell death (ICD) is regarded as a kind of programmed cellular death that offers momentous theoretical principle for immunotherapy of malignancies [43, 44]. The insufficient CDT for ICD triggering often achieves unsatisfactory tumor immunotherapeutic effect. Herein, we have developed a facile, one-step strategy for synthesizing multifunctional Cu&Ce oxide nanospheres with mesoporous nanostructure (mCu&Ce). After ICG encapsulating and surface grafting of RGD peptide (mCu&Ce@ICG/RGD), this nanoplatform is reported as accurate osteosarcoma recognition and tumor microenvironment (pH = 6.5) triggering tempestuous ICG, Cu and Ce ions release (Scheme 1). After entering osteosarcoma tumor cells, mCu&Ce@ICG/RGD could effectively produce hyperthermia and sequentially boost •OH generation under NIR laser irradiation. The PTT/CDT synergistic tumor ablation would be realized both in vitro and in vivo. Meanwhile, both heat and amplified ROS substantially mediate efficacious tumor immunotherapy by provoking ICD that activate effective T cells generation for a systemic anti-osteosarcoma immune response. What’s more, the Cu&Ce based nanoplatform permits precise early diagnosis of osteosarcoma by NIR II fluorescence and magnetic resonance dual-model bio-imaging. In a word, our work engineers a facile Cu&Ce based nanoplatform with bimodal bio-imaging properties. It can specifically recognize osteosarcoma for effectuating cancerous cells suppression by PTT enhanced CDT, which further profoundly induces ICD enhancement.

## Materials and methods

### mCu&Ce synthesis

Here, mCu&Ce was synthesized for the first time by a facile hydrothermal method. In brief, 60 mL of deionized water containing 0.07 g of CuCl_2_ and 0.03 g of CeCl_3_ was stirred at 90 °C for 30 min, subsequently, 0.2134 g of urotropine power was added to above mixed solution and kept vigorous stirring for 5, 10, 20 and 30 min. Finally, mCu&Ce nanospheres were separated by centrifugation (3500 rpm, 5 min) and washed by deionized water for remove the residual urotropine. Thereafter, the obtained mCu&Ce was dried overnight at vacuum with 60 °C.

### mCu&Ce@ICG synthesis

Then mCu&Ce@ICG was fabricated as following: A mixed solution containing 3 mL, 2.5 mg/mL mCu&Ce (deionized water) and 3 mL, 200 µg/mL of ICG (deionized water) was prepared. Latterly, this solution was gently oscillated for 120 min (25 °C, 100 rpm). In the end, the precipitate was collected *via* centrifugation (3500 rpm, 5 min) and then, the product of mCu&Ce@ICG was obtained after three-times washing to separate the residual ICG.

### mCu&Ce@ICG/RGD synthesis

For surface amino group’s functionalization (mCu&Ce@ICG-NH_2_), a mixed solution containing 10 mL of deionized water, 100 mg of mCu&Ce@ICG and 25 mg of PEG_2000_-NH_2_ were magnetically stirred for 12 h at 37 °C. Then the precipitate was obtained by *via* centrifugation (3500 rpm, 5 min). For removing the residual PEG_2000_-NH_2_, the as-made product, mCu&Ce@ICG-NH_2_ was washed more than three-times by centrifugation (3500 rpm, 5 min). The final product was dried overnight and stored at 4 °C. Subsequently, 40 mg of N-(3-dimethylaminopropyl)-N′-ethylcarbodiimide hydrochloride (EDC) and 20 mg of N-hydroxysuccinimide (NHS) power were concurrently added to 10 mL of PBS solution (1X, pH = 7.4) containing 10 mg RGD. Then the mixture was continuously stirred overnight under room temperature. Then the prepared mCu&Ce@ICG/RGD was collected by centrifugation (3500 rpm, 5 min) and washed three-times using deionized water. Finally, the RGD anchored nanoplatform was re-dispersed in deionized water and stored at 4 °C.

### Intracellular ROS evaluation

Human-osteosarcoma cells (143b) were firstly seeded. After 24 h culturing, cells were pre-incubated with 100 µM of H_2_O_2_ (deionized deionized) for 2 h. Then the cell medium was removed and replaced with fresh medium containing mCu&Ce@ICG/RGD. After 12 h, an 808 nm laser irradiation was conducted (5 min, 1.5 W/cm^2^). Then the medium was removed and the tumor cells were washed twice by PBS. Thereafter, a fresh medium containing 1 µM of 2’,7’-dichlorodihydrofluorescein diacetate (DCFH-DA) was added and further incubated for 0.5 h. Subsequently, the cells were washed twice by PBS. The samples were imaged by confocal laser scanning microscope (CLSM, 488 nm excitation; 500-550 nm emission). Control 143b cells were directly treated with some Cu content nanoformulations, mCu@ICG/RGD, mCu&Ce@ICG/RGD and mCu&Ce@ICG/RGD plus laser shining were conducted by same procedure.

### Intracellular •OH evaluation

Cellular imaging of •OH was carried out at the same procedure of ROS assessment. After 12 h, cell medium of the above five groups was discarded and a fresh medium containing 2.0 µM of hydroxyphenyl fluorescein (HPF) was added and further incubated for 0.5 h. Finally, the above five groups were observed by CLSM (488 nm excitation; 500-550 nm emission).

### Intracellular HMGB1 evaluation

Cellular imaging of HMGB1 was carried out at the same grouping of ROS assessment. After 12 h incubation, cells from each group were washed with PBS for twice and fixed with 4% paraformaldehyde for membrane disruption. Then cell samples were co-cultivated with a HMGB1 secondary antibody which was labeled by FITC. Subsequently, CLSM was used to observing HMGB1 expression (488 nm excitation; 500-550 nm emission).

### Construction of animals models

BALB/c nude mice (female, 6-week-old) were obtained from Shanghai Slac Laboratory Animal Co., Ltd. And all animal experiments were studied under a protocol approved by the Institutional Animal Care and Use Committee of Fudan University. Subcutaneous osteosarcoma mice model was prepared by subcutaneously inoculated 143b cells (5 × 10^6^/mouse) on the right hind-limb of BALB/c nude mice. After 15 days injection, tumor volume size can reach to 200–300 mm^3^ for the subsequent bio-imaging. Popliteal lymph-node metastasis was built by intra-palm injection of 143b cells (1 × 10^7^/mouse) at the left rear sole. After 21 days injection, the metastasis can be used for the subsequent MRI.

### NIR II fluorescence bio-imaging of tumors in vivo

mCu&Ce@ICG/RGD and mCu@ICG/RGD were intravenously injected into the subcutaneous osteosarcoma mice, respectively. Then all the mice were immediately imaged by a NIR II fluorescence bio-imaging system (808 nm laser excitation, 1000 nm long-pass filter) at various post-injection time periods. Besides, the major organs and tumors tissues were resected at 24 h post-injection of the two groups and further analyzed by ex vivo NIR II fluorescent bio-imaging.

### MRI for popliteal lymph-node metastasis

mCu&Ce@ICG/RGD and Gd-DTPA dispersion were intravenously injected into lymph-node metastasis bearing mice, respectively. Then the mice were immediately imaged by the MRI system at various post-injection time periods.

### In vivo photothermal imaging

For photothermal imaging in vivo, mCu&Ce@ICG/RGD, mCu@ICG/RGD and PBS (control) were intravenously injected into subcutaneous osteosarcoma mice, respectively. After 24 h post-injection, then tumor sites were conducted a prolonged exposure by 808 nm light (1.5 W/cm^2^). Immediately, photothemral bio-images were photographed by a FOTRIC 225s IR camera.

### In vivo osteosarcoma tumor inhibited appraising

Osteosarcoma tumor-bearing nude mice were stochastically assigned into 6 groups (*n* = 5) when the tumor volume reached ∼ 100 mm^3^. Then mice were injected with the following formulations (PBS, L, mCu@ICG/RGD, mCu&Ce@ICG/RGD, mCu@ICG/RGD + L, and mCu&Ce@ICG/RGD + L) *via* vena caudalis. After 24 h post-injection, the last two laser groups was were conducted a prolonged exposure by 808 nm laser (1.5 W/cm^2^, 5 min). Then, tumor size of length & width, body weight were measured at every 3 days during the whole 21 days of treatment, in which the final tumor volume values were calculated by the following formula: tumor volume = length ×width^2^/2. At the last day, the tumor tissues of 3 mice were resected and stained by using hematoxylin and eosin (H&E), terminal deoxynucleotidyl transferase dUTP nick end labeling (TUNEL) and Ki67 antibody, respectively. Meanwhile, major organs were also dissected and then stained by H&E.

### In vivo ICD evaluation

To examine ICD effect after different treatments, osteosarcoma tumor-bearing Balb/c mice were stochastically assigned into 5 groups (*n* = 5) and treated with different formulations (PBS, L, mCu@ICG/RGD, mCu&Ce@ICG/RGD, mCu@ICG/RGD + L, and mCu&Ce@ICG/RGD + L). After 7 days, tumors and tumor-drained lymph nodes were resected for single-cell suspension collecting. The cell suspension of the tumor-drained lymph nodes were co-stained with anti-mouse CD86-PE, CD80-APC, and CD11c-FITC and then analyzed using flow cytometer. Moreover, cytotoxic T lymphocytes (CTLs) and helper T cells in tumors were co-stained with anti-mouse CD8-PE, CD4-FITC, and CD3-APC, and then quantitatively analyzed.

## Results and discussion

### mCu&Ce@ICG/RGD construction and characterization

**Fig. 1:**
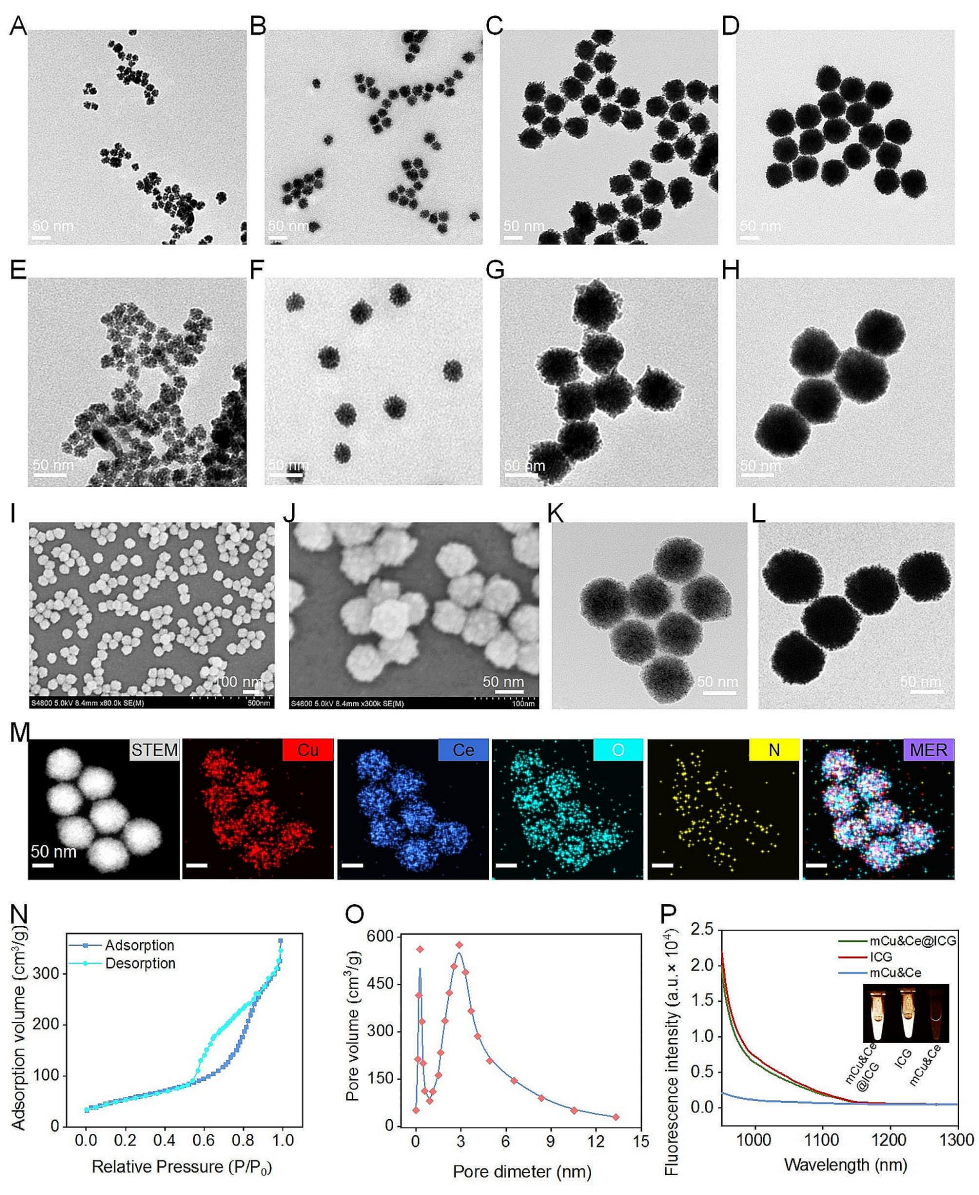
Large-scale and magnified TEM photographs of mCu&Ce after added urotropine for 5 min (**A**, **E**), 10 min (**B**, **F**), 20 min (**C**, **G**), 30 min (**D**, **H**). (**I**) Large-scale and (**J**) magnified SEM photographs of mCu&Ce with urotropine adding for 20 min. (**K**) HRTEM image of mCu&Ce. (**L**) TEM image of mCe&Cu@ICG/RGD. (**M**) Cu, Ce, O, N and merged elemental images of mCe&Cu@ICG/RGD. (**N**) Nitrogen adsorption/desorption curve of mCe&Cu. (**O**) Pore size distribution of mCe&Cu. (**P**) Emission spectra and NIR II luminescence images (insert) of mCe&Cu@ICG/RGD, ICG and mCe&Cu.

The exact procedure for the fabrication of mCu&Ce@ICG/RGD nanoplatform is displayed in Scheme 1. In brief, the hydrophilic mCu&Ce nanoparticles are prepared for the first time with copper chloride (CuCl_2_) and cerium chloride (CeCl_3_) as the precursors (weight ratio = 7:3) under an aqueous system. After homogenous stirring under 90 °C, subsequently, a series of alloyed Cu&Ce nanospheres with rough surface can be obtained after introducing urotropine for various periods. As illustrated in Fig. 1A-D, the diameters distinctly increase from 20 to 78 nm when the incubation time prolongs 5 to 30 min (Fig. 1E-H), concurrently, aqueous dispensability and the crystallinity degree of Cu&Ce nanospheres also dramatically raise as the processing time extends. Meaningfully, CuO nanoleaves with lamellar staked mesopores (mCu) (Fig. S1) and CeO_2_ nanogranules (Fig. S2) can be obtained when only Cu and Ce based precursors are introduced, respectively. Amorphous Cu&Ce based nanogranules stack and assemble into spherical nanoparticles under the observation of Transmission electron microscope (TEM), especially when urotropine is added for 10 min, smaller Cu&Ce oxide nanoclusters (∼ 5 nm) are evidently detected. Interestingly, according to previous work [45], stacked mesopores generate during the two building blocks pile up that considerably decrease over the incubation time. Considering the deceased surface roughness, pore size and the increased diameter of the prepared bimetallic oxide nanoparticles with mesoporous nanostructure (mCu&Ce), the group of 70 nm (20 min) is finally chosen for the following in vitro and in vivo studies. Additionally, it is also characterized by scanning electron microscopy (SEM), notably, monodispersed mCu&Ce nanospheres with rough interface can be plainly visualized (Fig. 1I, J). According to the TEM photograph with high resolution (HRTEM), consequently, randomly dispersed ultrasmall nanogranules can be clearly observed in the prepared bimetallic oxide nanospheres (Fig. 1K). Moreover, high-angle-annular dark field SEM (HAADF-STEM) is employed for the elemental mapping of mCu&Ce. Both the elemental mapping pictures and energy-dispersive X ray spectra (EDS) show the co-existence of Cu, Ce, O and N elements (Fig. 1M, S3), profoundly confirming the Cu&Ce co-doped nanocomposite is easily and successfully synthesized. This nanocomposite is further characterized by the X-ray photoelectron spectrocopy (XPS). As illustrated in Fig. S4A, two characteristic peaks of Cu 2p (Cu^2+^) appear at 934.8 eV (2p_2/3_) and 954 eV (2p_1/2_), meanwhile, Ce 3d in XPS spectra indicates the mix-valence of Ce^3+^ and Ce^4+^ states and Ce^3+^/Ce^4+^ ratio in mCu&Ce is 0.45 (Fig. S4B). Higher levels of Ce^+ 4^ state presents more Fenton-like activity for the ROS generation. The nitrogen adsorption/desorption isotherm of mCu&Ce is appraised at 77 K. As depicted in Fig. 1N, O, the specific surface area is calculated to be approximately ∼ 251 m^2^g^− 1^ and the diameter of the stacked mesopores is measured in range of 0.2 ∼ 2.8 nm. The relatively large specific surface area and pore size are vitally important for the effective delivery of therapeutics. Further, the first-line clinical fluorophore, ICG is loaded into the mesopore nanostructure (mCe&Cu@ICG) with the loading efficiency of ∼ 12.5% (w/w). Next, for prolonging blood circulation time and the subsequent targeting motifs modification, the hydrophilic PEG_2000_-NH_2_ is wrapped on the interface of mCe&Cu@ICG. Finally, the activate osteosarcoma recognition ligand, RGD is cross-linked on the outlayer of ICG loaded bimetallic nanoparticles *via* dehydration condensation reaction (mCe&Cu@ICG/RGD). Exhilaratingly, ζpotential distinctly decreases after surface grafting of RGD, which could be ascribed to the consumption of -NH_2_ groups (Fig. S5). Unobvious morphological transformation and size variation are discovered in mCe&Cu@ICG/RGD (Fig. 1L, S6). In the meantime, analogous with ICG, the emission spectra of ICG encapsulated nanoplatform extends ideally into NIR II and the off-peak NIR II luminescence images of above two samples are quite strong to justify the successful engineering of mCe&Cu@ICG/RGD (Fig. 1P).

### pH sensitive biodegradation, ROS production and hyperthermia determination

**Fig. 2:**
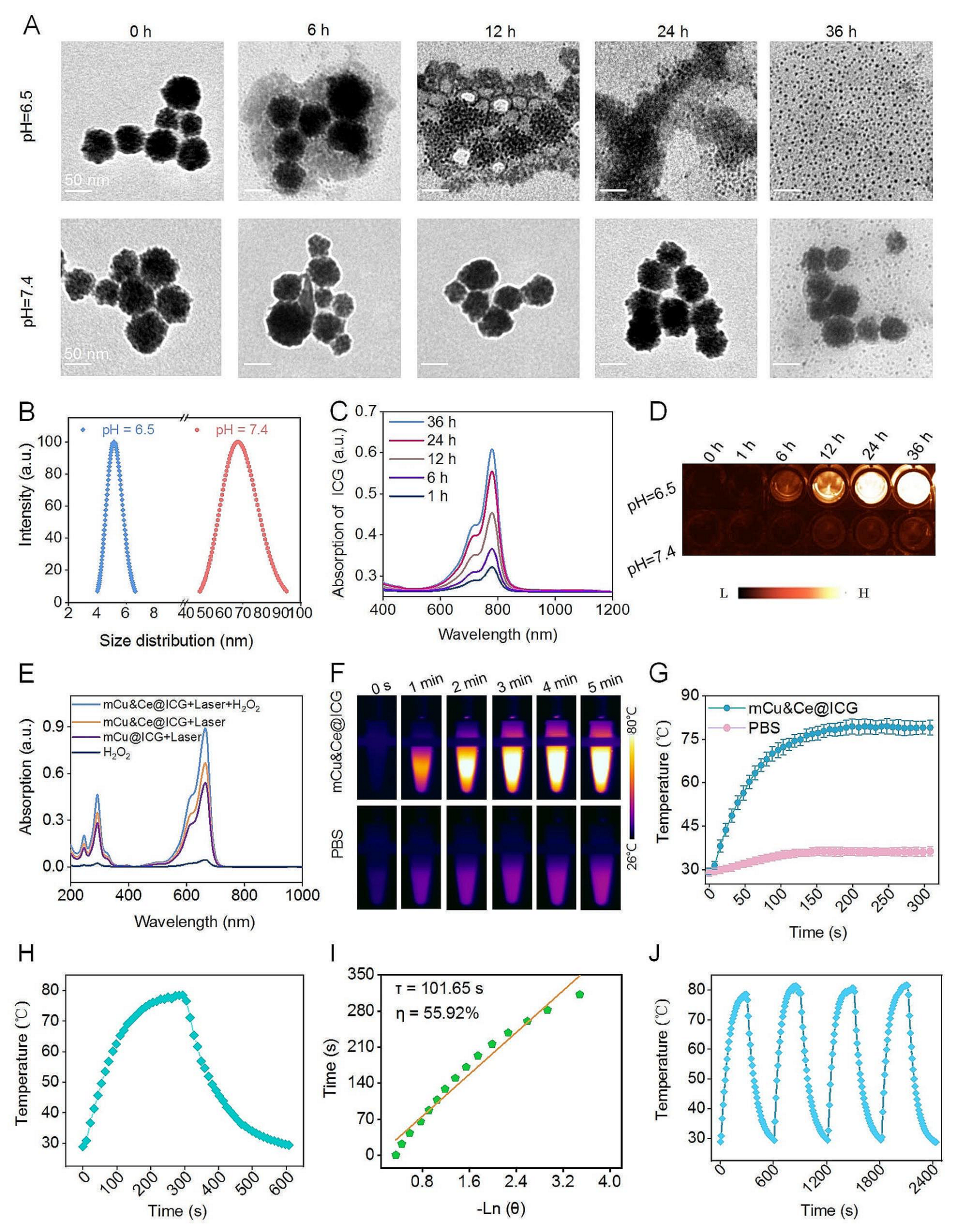
(**A**) TEM-images of mCe&Cu@ICG/RGD after incubated with biological buffers at pH = 6.5 and pH = 7.4 for different times. (**B**) Average diameter variation of mCe&Cu@ICG/RGD before or after treated with pH = 6.5 buffer for 36 h. (**C**) The absorption spectra and (**D**) NIR II fluorescent images of released ICG from the supernatant of pH = 6.5 treated mCe&Cu@ICG/RGD for various hours. (**E**) UV-*vis* spectra of oxide TMB from the supernatant of various formulations under weak acid treatment. (**F**) Hyperthermia pictures, (**G**) temperature rose profiles of mCe&Cu@ICG/RGD and PBS plus laser irradiation for 300 s. (**H**) The thermal heating line of mCe&Cu@ICG/RGD under laser illumination for 300 s and the corresponded cooling curve after stopped the light exposure. (**I**) Linear relationship between cooling time and –Ln (θ). (**J**) Temperature changes of mCe&Cu@ICG/RGD under four cycles of laser irradiation and laser off

Since mCe&Cu@ICG/RGD is deliberately designed with the intention of activate ICG release, corresponded Fenton like reaction through mCe&Cu based framework biodegradation under extra-cellular weak acid triggering is further determined. Owing to the stacking force of bimetallic oxide nanogranules is fragile under the immersion of tumor microenvironmental conditions [45], the morphological transformation of mCe&Cu@ICG/RGD is recorded by TEM after treated with various biological relevant buffers, including normal tissue (pH = 7.4) and tumor stroma tissue (pH = 6.5) (Fig. 2A). Interestingly, the biodegradation efficiency under pH = 6.5 condition treatment is prominently higher than that of pH = 7.4 group at all-time points with preliminary framework collapse and nanogranules release at 6 h, and all nanospheres disappear with substantial Cu&Ce based granulum emerging at 36 h. These nanogranules are capable of being conductive for tumor tissue infiltration. Exhilaratingly, the morphology is constantly stable over 24 h immersion of normal condition and only trace number of small nanoparticles discharge at 36 h treating (Fig. 2A). Interestingly, the average diameter of mCe&Cu@ICG/RGD drops sharply from ∼ 68 nm to ∼ 5 nm after 36 h immersion of extra-cellular weak acid pH value in tumor tissue that further manifesting the whole structural disintegration. Concurrently, the released profile of ICG in the supernatant of pH = 6.5 physiological buffer is also determined in a variety of incubation periods. We observe that ICG dyes are gradually discharged with a time-dependent manner (Fig. 2C). Simultaneously, as illustrated by NIR II luminescent images of released free ICG in pH = 6.5 condition, fluorescent signal is pronouncedly enhanced within 36 h, remarkably stronger that of pH = 7.4 group (Fig. 2D). At the same time, similar released trends of Cu and Ce ions are demonstrated under the incubation of tumor microenvironmental stimulated buffer for various periods. Particularly, approximately 90% of Cu/Ce ions are discharged at 36 h incubation (Fig. S7). Collectively, all the fluorescent intensity changing and ions release trends of ICG are well in accordance with the framework detachment observation by TEM imaging. Concurrently, after treated for 36 h under weak acid environment, the Fenton-like catalysis effect of Cu&Ce ions is then evaluated by a commercial •OH indicator, 3,3’,5,5’-tetramethylbenzidine (TMB). Under the catalyzing of •OH, the product, oxide TMB possesses three characteristic peaks, obviously, in contrast with mCe&Cu@ICG/RGD + L group, mCu@ICG/RGD shows only marginal ROS productivity, as expectedly, mCe&Cu@ICG/RGD + H_2_O_2_ + L exhibits 2-fold higher •OH increasing. XPS data further confirms the change of both Cu and Ce state after treatments of H_2_O_2_ plus laser irradiation. The original satellite peak (944.8 eV) in Cu 2p disappears and the ratio of Ce^3+^/Ce^4+^ increases to 1.03 substantially prove that Cu^2+^ and Ce^4+^ turn into Cu and Ce^4+^ after photothermal boosted Fenton-like reaction (Fig. S8). Also, as shown in the electron paramagnetic resonance (ESR) characteristic spectra (Fig. S9), a considerable amount of •OH is generated after the addition of H_2_O_2_ to mCe&Cu@ICG/RGD aqueous solution under laser illumination compared with PBS and mCe&Cu@ICG/RGD groups under high H_2_O_2_ conditions. All these findings primarily reveal the advantage of bimetallic Fenton-like nanoagents and the augmented chemodynamic capability of our nanoplatform plus 808 nm light illumination under high H_2_O_2_ condition (Fig. 2E). Subsequently, as the strong 808 nm laser absorption of ICG that endows mCe&Cu@ICG/RGD with potent photothermal conversion performance. As displayed in Fig. 2F, G, the temperature of nanoplatform exhibits an obvious time related rise and it climbs to the maximum level (79.1 °C) under continuous 808 nm laser exposure for 300 s, testifying a quick NIR light response. In marked contrast, the temperature in PBS solution ascends slightly under the same treatment with only 36.3 °C in the endpoint of laser irradiation. Additionally, to further detect the laser-to-thermal conversion efficiency (η), the heat discrepancy of mCe&Cu@ICG/RGD dispersed in aqueous solution is latterly calculated from healing-to-cooling cycle (Fig. 2H). Based on the previous formula, the concrete η value is determined approximately as ∼ 55.92% (Fig. 2I), evidently, it is equivalent to the reported inorganic nanocarriers with dendritic mesopores for ICG delivery [44]. Meanwhile, exceptional photothermal stability is also monitored after four 808 nm laser on-off cycles (Fig. 2J). On the whole, all results confirm that the tumor responsive programmed mesoporous Cu&Ce nanocarriers with ICG loading can be further applied for malignancy suppression *via* PTT-CDT.

### In vitro cell killing *via* PTT-CDT and expression of ICD indictors

**Fig. 3:**
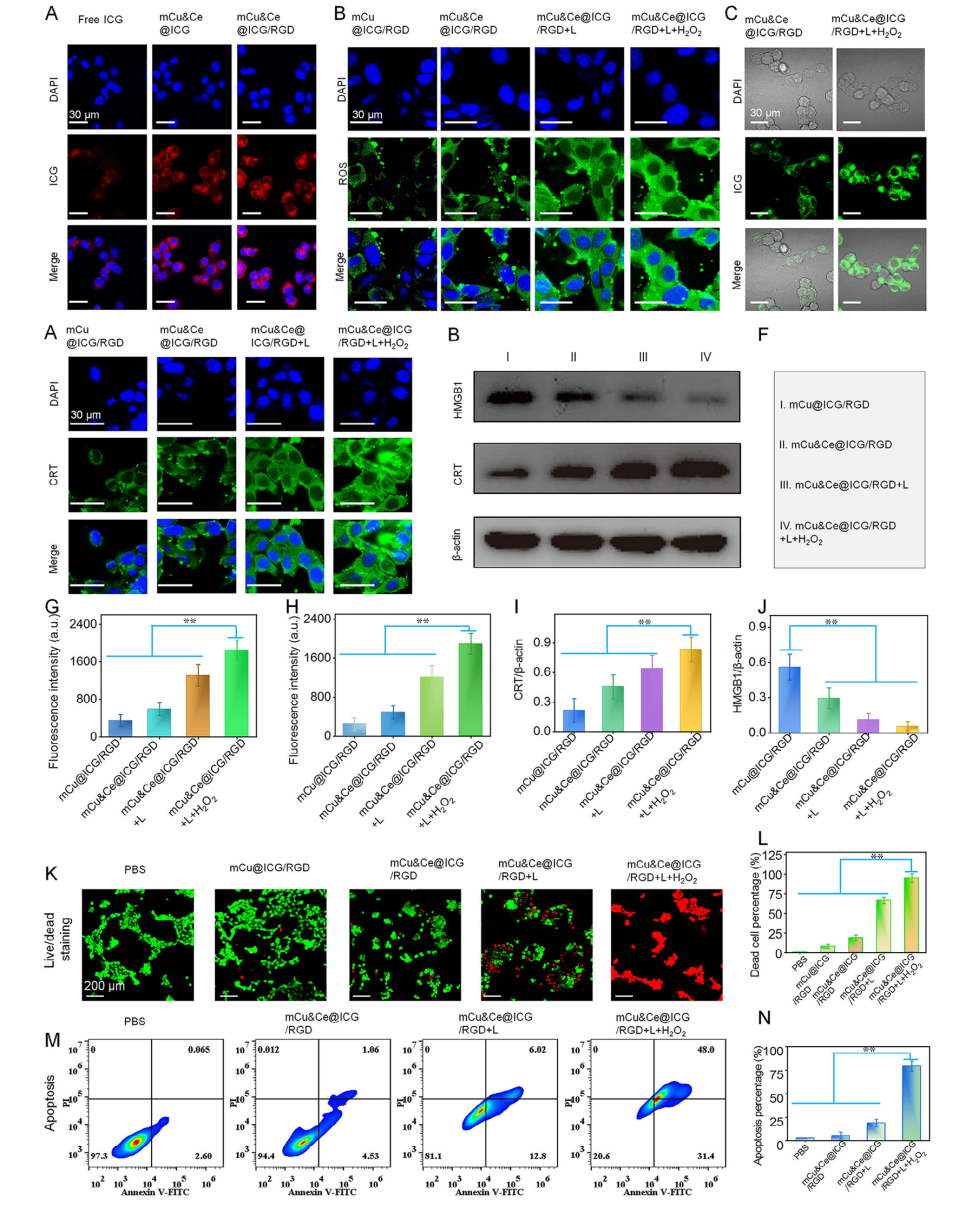
(**A**) CLSM photographs of 143b cells after treated with free ICG, mCu&Ce@ICG and mCu&Ce@ICG/RGD. (**B**) ROS measurement images of 143b cells after incubated with mCu@ICG/RGD and mCu&Ce@ICG/RGD followed by laser irradiation and H_2_O_2_ + laser. (**C**) CLSM pictures of intracellular •OH production after 143b cells were treated with mCu&Ce@ICG/RGD and mCu&Ce@ICG/RGD + H_2_O_2_ + L, respectively. (**D**) CLSM images of membrane-anchored CRT expression in 143b cells after incubated with mCu@ICG/RGD and mCu&Ce@ICG/RGD followed by laser irradiation and H_2_O_2_ + laser. (**E**, **F**) Western-blot analysis of CRT and HMBG1 expressions in 143b cells after incubated with mCu@ICG/RGD and mCu&Ce@ICG/RGD followed by laser irradiation and H_2_O_2_ + laser. (**G**) Quantitative green signal intensity in cellular uptake in Fig. 3A. (**H**) Quantitative green signal intensity of ROS generation in Fig. 3B. The ratios of CTR/β-action (**I**) and HMGB1/β-action (**J**) in Fig. 3E. (**K**) live-dead cell fluorescent images, (**M**) Cell apoptosis-necrosis analysis and (**L**, **N**) corresponded quantitative analysis of 143b cells after treated with various formulations. **p < 0.01. Above presented data is as means with standard deviations (*n* = 3) (mean ± SD)

For nanoplatform based drug delivery system, it is conductive to accurately target and strongly promote intracellular uptake of multi-mode therapeutics. Firstly, according to the CCK-8 kit for cell viability assessment, over 90% of 143b cells still alive even the concentration of nanoplatform is set as high as 300 µg/mL, confirming the exceptional bio-compatibility of the therapeutic agents (Fig. S10). To investigate the cellular endocytosis behavior of mCe&Cu@ICG/RGD, the internalization effect was firstly determined by a CLSM. As illustrated in Fig. 3A, the red light of ICG treated with RGD modified nanoplatform was significantly stronger than that of mCe&Cu@ICG and free ICG. Clearly, we inspect that both α_v_ and β_3_ subunits are expressed by 143b cells that remarkly higher than normal cells (Fig. S11). The enhanced cellular internalization of mCe&Cu@ICG/RGD might be contributed to RGD peptide which could specifically recognize α_ν_β_3_ integrin that overexpressed on cytomembrane of osteosarcoma cells. Here, both Cu and Ce can catalyze the decomposition of H_2_O_2_ to greatly increase the intratumoral level of lethal ROS. Subsequently, the free-radical level in cytoplasm is further evaluated. Here, DCFH-DA was applied to act as a fluorescence probe for verifying the in situ intracellular ROS generation. As presented in Fig. 3B, in contrast to mCu&Ce@ICG/RGD group, dim green fluorescence was visualized in mCu@ICG/RGD group, this could be ascribed to the low biodegrade rate of the former one. Relatively low Cu ions is discharged from mCu nanoleaves with lots of Cu based fragments are found in 36 h incubation of pH = 6.5 buffers (Fig. S12). Notably, in accordance with ROS generation trend in bulk solution, it is significantly intensified when the 808 nm light exposure is employed accompanying with H_2_O_2_ pre-treating (Fig. 3G). The findings reveal that higher thermal generation could substantially augment Fenton like reaction for the consequence of ROS boosting highlight the significance of our study. Both hyperthermia and generated ROS during the laser irradiation can trigger detrimental bio-chemical changes, the most possible disruption is the alternation of the mitochondria membrane potential (MMP) [46], so we next inspect the mitochondrial dysfunction of 143b cells after stained with a commerial dye of JC-1. Sharply, mCu&Ce@ICG/RGD + H_2_O_2_ + L mediates to an significant MMP loss because of the strongest green fluorescence (Fig. S13). This findings illustrate well that our nanoplatfrom induces apoptotic/necrototic cells is probably through the pathway with mitochondrial dependence. Furthermore, we performed whether mCu&Ce@ICG/RGD can mediated •OH amplification in cytoplasm after being stained by a frequently-used indicator, HPF. Obviously, comparable higher (5.3 folds) green luminescence is visualized in 143b cells administrated with mCu&Ce@ICG/RGD + H_2_O_2_ + L. ICG can also be act as a photodynamic sensitizer, mesoporous silica loaded ICG with RGD modification (MSN@ICG/RGD,70 nm) is applied for ROS generation under 808 nm lser irradiaiton (Fig. S14). Unfortunately, negligible ROS level is found in cytoplasm (Fig. S15), verifying that Fenton-like catalysis effect of Cu&Ce ions are conductive to cancerous cell killing.

Owing to high oxidative stress could massively evoke ICD, high translocation of calreticulin (CRT) from endoplasmic reticulum to the cytomembrane can be detected. It is capable of stimulating both macrophage and immature-dendritic cells (DCs) to engulf dying cells and debris. Besides, in the surroundings of dying tumor cells, extracellular high mobility group protein B1 (HMGB1) release is another important feature of the ICD-corresponded immunogenicity. Therefore, the reduced HMGB1 level in cytoplasm also plays a critical role in ICD. We have also apprised the membrane anchored CRT by CLSM. As illustrated in Figs. 3D and 143b and b cells treated with mCu&Ce@ICG/RGD + H_2_O_2_ + L mediate highest levels of CRT in contrast with other formulations, which is agreed with intracellular ROS amplified results. Additionally, the diminished HMGB1 signal is demonstrated in this group, this opposite trend of CRT level further manifesting the amplified ICD effect of our nanoplatform (Fig. 3D). Subsequently, to further illustrate the expression of ICD-related proteins, the CRT and HMGB1 levels in 143b after various treatments are studied by western-blot assay. Clearly, CRT is significantly up-regulated on cellular membrane and HMGB1 is dramatically down-regulated in cytoplasm when 143b cells are treated with mCu&Ce@ICG/RGD + H_2_O_2_ + L (Fig. 3E, F). In comparison with mCu&Ce@ICG/RGD group, above expressed protein levels are approximately two-fold higher and five-fold lower in mCu&Ce@ICG/RGD + H_2_O_2_ + L, respectively (Fig. 3I, J), revealing the powerful ICD provoking capability of this treatment. Finally, live-dead staining images and cellular apoptosis-necrosis studies are obtained by CLSM and flow cytometer, respectively. Analogous with intracellular ROS generation and HMGB1 results, 143b cells experience most efficacious cell death in mCu&Ce@ICG/RGD + H_2_O_2_ + L (Fig. 3K-N). As expected, when the concentration of mCu&Ce@ICG/RGD increases to 300 µg/mL cell viability of 143b cells in H_2_O_2_ pre-incubation plus laser illunimation group is only half of pure nanoplatform treated group (Fig. S17). This highest tumor cell killing is primarily mediated by PTT simultaneously amplified both ROS and ICD.

### In vivo tumor targeting evaluation by fluorescent imaging, MRI and photothermal assessment

**Fig. 4:**
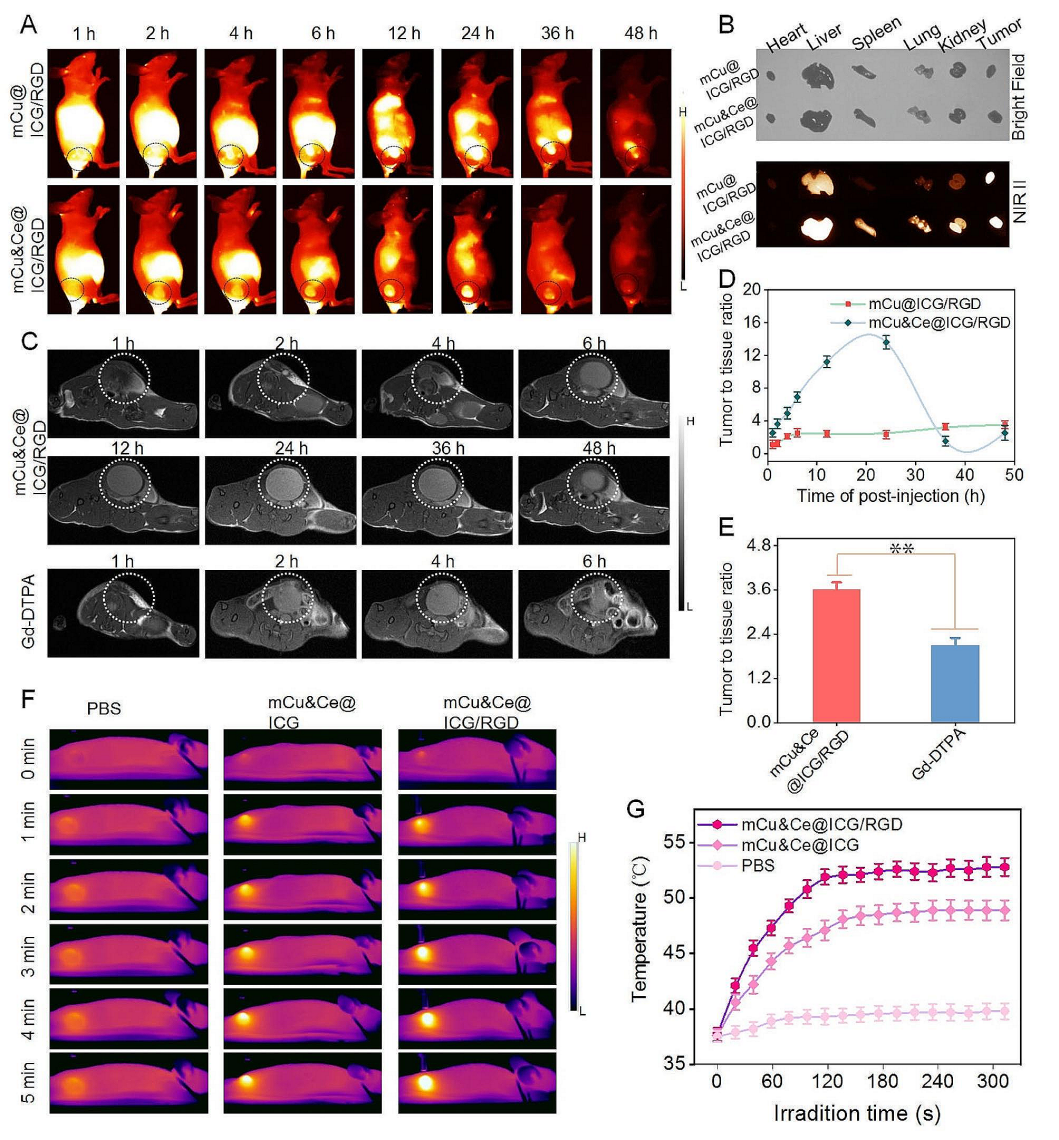
(**A**) NIR II fluorescent bio-images of subcutaneous osteosarcoma bearing mice after injection of mCu@ICG/RGD or mCu&Ce@ICG/RGD *via* vena-caudalis at various time intervals. (**B**) Ex vivo fluorescent image of resected tumor and major organ tissues from above two groups at 24 h postinjection. (**C**) T1-weighted MRI bio-images of tumor bearing mice after vena-caudalis administration of mCu&Ce@ICG/RGD and Gd-DTPA for various periods. (**D**) Tumor to tissues ratio of NIR II fluorescence imaging and (**E**) Tumor to tissues ratios of mCu&Ce@ICG/RGD and Gd-DTPA. (**F**) Photothermal bio-images and (**J**) corresponded temperature increased curves in osteosarcoma tumor site after continuously irradiated by 808 nm laser for different seconds postinjecting of PBS, mCu&Ce@ICG, and mCu&Ce@ICG/RGD. **P < 0.01

Considering the pleasing cellular recognizing effect in vitro, we are inspired to investigate the bio-distribution and tumor concentrated behavior of mCu&Ce@ICG/RGD against osteosarcoma tumor-bearing nude mouse model. Firstly, to obtain accurate tumor contour discrimination, mCu@ICG/RGD and mCu&Ce@ICG/RGD are intravenously injected into subcutaneous bearing mice, respectively. Immediately, NIR II fluorescence bioimages are photographed at specific periods to monitor the tumor targeting and biodistribution in vivo of our nanoplatform *via* a small animal NIR II fluorescence imaging bio-system. Evidently, the outline of tumor can be gradually delineated with the fluorescent signal (beyond 1000 nm) initially concentrates in the tumor site at 2 h post-injection of mCu&Ce@ICG/RGD. Then it reaches to the maximum level at 24 h with tumor contour distinctly discriminating from the surrounding peripheral muscle tissue; subsequently, it decays slowly over time with residual nanoplatform maintains at 48 h (Fig. 4A). While the fluorescent signal of mCu@ICG/RGD is mainly scattered in the liver and prominently higher than mCu&Ce@ICG/RGD group at all-time intervals. Based on this high accumulation in liver, the tumor tissue of the latter group is hardly to distinguish (Fig. 4A). Meanwhile, tumors and main organs are harvested for ex vivo NIR II fluorescent bio-imaging. Notably, even though a comparative light signal intensity in tumors of above two groups is visualized, the mCu&Ce@ICG/RGD treated liver possesses prominently lower intensity than that of mCu@ICG/RGD (Fig. 4B). Here, the relatively rapid biodegradation behavior of the former group is conductive to the liver clearance. Accordingly, tumor to surrounding normal tissue ratio is calculated by the semi-quantitative mean NIR II signal intensity. mCu&Ce@ICG/RGD presents six folds higher value than that of mCu@ICG/RGD at 24 h postinjection (Fig. 4D). Further, we have also verified the specific tumor recognition of our Cu based nanoplatform by MRI with clinical Gd-DTPA as the control. According to the sequential T1WI MRI bioimages at various intervals, MRI signal of osteosarcoma bearing lymphatic metastasis with plantar injection of mCu&Ce@ICG/RGD tremendously increases to the peak level in 24 h postinjection, and from this time-point on, it gradually decays to the basal intensity (Fig. 4C). Nevertheless, owing to the rapid excretion of Gd-DTPA, the highest tumor accumulation can be discovered at 2 h postinjection. Tumor to tissue ratio of our nanoplatform at 24 h is remarkably higher than that of Gd-DTPA (Fig. 4E), further demonstrating the effective tumor-targeting capability of mCu&Ce@ICG/RGD and the laser irradiation for PTT must be conducted at this time. In the end, in vivo photothermal conversion effect in the subcutaneous osteosarcoma bearing mice is investigated after tail vein injection of PBS, mCu&Ce@ICG and mCu&Ce@ICG/RGD. Specifically, the temperature in the nanoformulation treated tumor site changes sharply and increases up to the peak value (48.9 and 52.8 °C, respectively) with maximum photothermal maintaining (Fig. 4F, G). Undoubtedly, this phenomenon is mainly ascribed to the active targeting ability of RGD modification. For PBS treated mice, the temperature only shows slightly increase (39.8 °C) even after 300 s illumination (Fig. 4F, G). Consequently, above in vivo bio-imaging results highlight the promising multimode contrast nanoagents for tumor diagnosis and satisfied hyperthermia performance for tumor suppression.

### In vivo PTT CDT and ICD evaluation

**Fig. 5:**
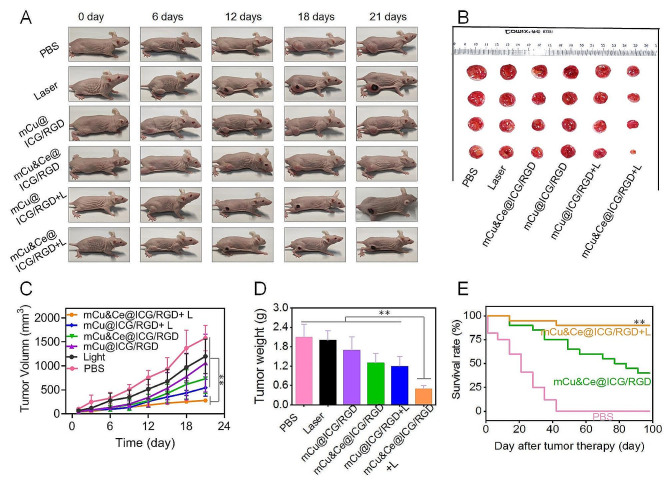
Digital photographs of (**A**) subcutaneous osteosarcoma bearing mice and (**B**) the totally resected tumors after various formulations treating for 21 days, respectively. (**C**) Representative tumor volume changes during the whole treatment and (**D**) dissected tumor tissues at the last day. (**E**) Life-span curves after various administration for 100 days. **P < 0.01

Based on above promising in vitro cell killing and exceptional tumor accumulation of our Cu&Ce based nanoplatform, a 143b tumor xenograft-mice model is established to further investigate the in vivo PTT/CDT/ICD synergistic therapeutic efficacy of mCu&Ce@ICG/RGD. To validate our programmed therapy hypothesis, subcutaneous osteosarcoma bearing mice are administrated with six different formulas (PBS, L, mCu@ICG/RGD, mCu&Ce@ICG/RGD, mCu@ICG/RGD + L, and mCu&Ce@ICG/RGD + L). As depicted in Fig. 5A-D, the tumor tissues of mice with treatment of PBS or laser grow rapidly throughout the whole treatment, confirming that the 808 nm laser (5 min, 1.5 W/cm^2^) alone has little suppression effect toward tumor growth. Expectedly, as compared to mCu@ICG/RGD with partial ablated effect, owing to faster biodegradable rate, relatively higher tumor growth inhibition is found to those treated with mCu&Ce@ICG/RGD, comparatively, both tumor volume and tumor weight in nanoparticles plus laser irradiation groups are noticeably controlled. Intriguingly, mCu&Ce@ICG/RGD + L administrated tumors are basically suppressed with strikingly lower tumor inhibition rate in comparison with other groups. Clearly, this exhaustive eradiation efficiency might be attributed to the synergistic PTT-augmented ROS amplification. As a result, mCu&Ce@ICG/RGD administration upon laser irradiation can prominently extend the life-span with over 90% of cured mice living more than 100 days, contradistinctively, all mice with PBS treatment dead within 42 days (Fig. 5E), profoundly demonstrating that our Cu&Ce based synergistic therapy of PTT-CDT possesses the best tumor suppression performance.

**Fig. 6:**
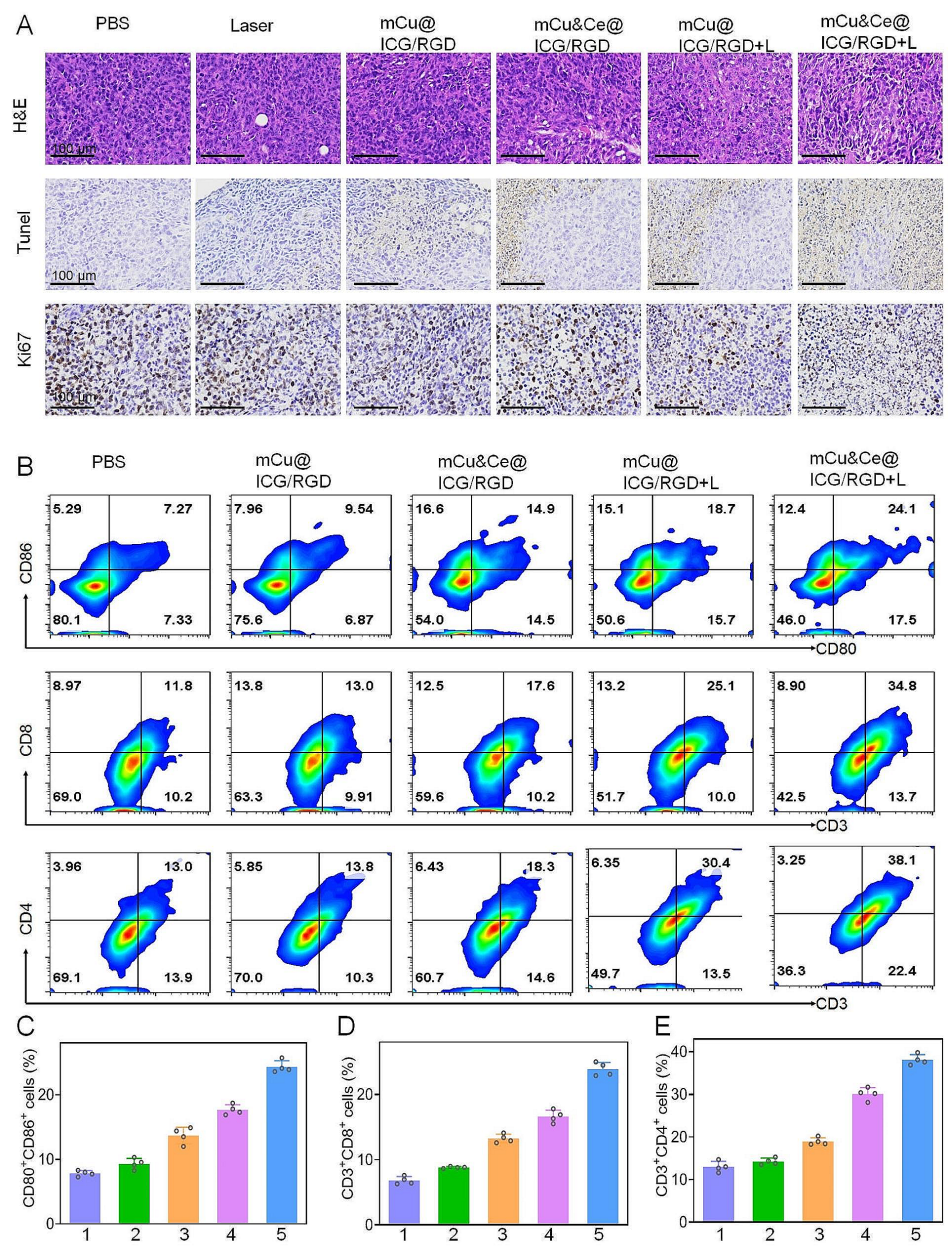
(**A**) Ex vivo tumor inhibited evaluation after 21 days of various treatment *via* H&E and Tunel and Ki67 staining. Flow cytometer analysis of (**B**) DCs maturation (CD80^+^&CD86^+^) in tumor-drained lymphatic node, CTLs (CD8^+^&CD3^+^) and helper T cells (CD4^+^&CD3^+^) in tumor tissues. Quantitative percentage of above DCs (**C**), CTLs (**D**) and helper T cells (**E**). 1–5 refers to PBS, mCu@ICG/RGD, mCu&Ce@ICG/RGD, mCu@ICG/RGD + L, and mCu&Ce@ICG/RGD + L, respectively

Simultaneously, tumor tissues are also collected for H&E, Tunel and Ki76 staining studies. As illustrated in Fig. 6A, the most condensed nucleus chromatin and largest area of necrotic tumor cells can be observed in the H&E staining photograph of mCu&Ce@ICG/RGD + L administrated group. According to the immunohistochemical analysis of Tunel, this treatment also causes a significantly apoptotic/necrotic level in sharp contrast with other groups. In the barging, as a well-regarded cell proliferation indicator, the expression of Ki67 is also carefully investigated. Distinguished from control group and other nanoformulations, it macroscopically and substantially reduces in the last group, indicating that mCu&Ce@ICG/RGD + L exhibits strongest synergistic anticancer capability. Body-weight profiles from all groups demonstrate undiscernible fluctuations and unnoticeable histopathological abnormality is discovered in the major organs from Cu-based nanoplatform accompanying with laser illumination, implying the outstanding biocompatibility and ignorable systematic side-effects (Fig. S18, S19). Finally, tumor immunogenicity provoking *via* tumor-related antigen recognition and the followed presentation is crucial for ICD effect. In our study, both hyperthymia and amplified intracellular ROS play vital factor for ICD during which dying tumor cells have excreted the tumor-related antigens to provoke the DCs maturation and facilitate the infiltration of CTLs [44]. For the following ICD assessment, we have re-conducted tumor inhibited studies in osteosarcoma bearing Balb/c mice for one week. As the hallmarks of DCs maturation, CD80 and CD86 co-stimulated molecules in tumor-drained lymph node is apprised by flow-cytometer. Exhilaratingly, the percentage of CD80^+^&CD86^+^ DCs in mCu&Ce@ICG/RGD + L group (∼ 24.1%) is remarkably higher than other four groups (Fig. 6B, C). Meanwhile, CTLs in tumor tissue after various administrations are further assessed (CD3^+^&CD8^+^), obviously, compared with other groups, CD8^+^ T cells for tumor cell killing through cytotoxins releasing in the last group reaches the largest level (Fig. 6B,D). Besides, the adaptive immune response tends to be polarized which often involves CD4^+^ T cells (helper T cells) subsets. Unsurprisingly, the mice administrate with mCu&Ce@ICG/RGD + L exhibit a pronouncedly increased CD4^+^ T cells (Fig. 6B, E). ICD could not activate immune system due to the immunosuppressive tumor microenvironment. Here, we speculate that the degraded small Ce&Cu based nanoganules can also deplete intracellular GSH levels in partial [47, 48]. The GSH-consumption nanoplatform reprogram**s** tumor immunosuppressive microenvironment that strengths the ICD effect in our work. Simultaneously, pro-inflammatory cytokines in serum (such as: IL-2, TNF-α, and IFN-γ) of mCu&Ce@ICG/RGD + L treated mice significantly amplify in sharp contrast with other groups (Fig. S20), testifying the highest ICD levels in the body. Collectively, all data proves that our nanoplatform plus laser irradiation can potently activate ICD that could ultimately dictate the progression and regression of osteosarcoma metastasis.

## Conclusion

In summary, we have designed and successfully prepared a fascinating nanoplatform composed of mesoporous Cu&Ce oxide nanosphere for CDT and MRI, loaded ICG for NIR II contrast agent and PTT, as well as RGD for targeting motif. The promising nanotherapeutic is featured with unparalleled advantages like precise recognition for osteosarcoma tissue, NIR II fluorescent bio-imaging and MRI for tumor contour distinguishing and programmed anticancer performance through PTT evaluated CDT along with the activated ICD. The therapeutic effect is confirmed by efficaciously inducing cancerous cell death in vitro as well as powerfully eradicating the solid osteosarcoma in vivo with the prominent survival-rate prolonging. Moreover, the excellent biosafety performance is also manifested in vivo. Taken together, our work develops a unique paradigm for promoting targeted diagnosis and therapy for clinical malignancies.

### Electronic supplementary material

Below is the link to the electronic supplementary material.

Supplementary Material 1

## Data Availability

No datasets were generated or analysed during the current study.
