# Supplementary material for: Osteosarcoma-targeted Cu and Ce based oxide nanoplatform for NIR II fluorescence/magnetic resonance dual-mode imaging and ros cascade amplification along with immunotherapy
Source: J Nanobiotechnology. 2024 Apr 4;22:151. doi: 10.1186/s12951-024-02400-z (PMC10993435; doi:10.1186/s12951-024-02400-z)
Supplement: Supplementary file 1 — Supplementary Material 1 [file 12951_2024_2400_MOESM1_ESM.docx]

**Osteosarcoma-targeted Cu and Ce Based Oxide Nanoplatform for** **NIR II Fluorescence/Magnetic Resonance Dual-mode Imaging and ROS Cascade Amplification Along with Immunotherapy**

Mo Cheng^1^, Qingjie Kong^4^, Qing Tian^5^, Weiluo Cai^1^, Chunmeng Wang^1^, Minjia Yuan^3,6^, Wenxing Wang^3, *^, Peiyuan Wang^2, *^, Wangjun Yan^1, *^

1. Department of Musculoskeletal Surgery of Shanghai Cancer Center, Fudan University, Shanghai 200032, P. R. China.
2. Key Laboratory of Design and Assembly of Functional Nanostructures, Fujian Institute of Research on the Structure of Matter, Chinese Academy of Sciences, Fuzhou 350002, P. R. China.
3. Department of Chemistry, *i*ChEM (Collaborative Innovation Center of Chemistry for Energy Materials), Shanghai Key Laboratory of Molecular Catalysis and Innovative Materials, State Key Laboratory of Molecular Engineering of Polymers, Laboratory of Advanced Materials, Fudan University, Shanghai, 200433, P. R. China
4. Department of Orthopedics, Shanghai General Hospital, Shanghai Jiao Tong University School of Medicine, Shanghai, 200080, P. R. China.
5. Department of Neurology，Qingpu Branch of Zhongshan Hospital, Fudan University, Shanghai, 201799, P. R. China
6. Shanghai Qiran Biotechnology Co., Ltd., Shanghai 201702, China

*Email Address of the Corresponding Author: yanwj@fudan.edu.cn, wangpeiyuan@fjirsm.ac.cn, wangwenxing@fudan.edu.cn

**Supplementary experimental section**

1. **Materials**

Copper nitrate hydrate (Cu(NO_3_)_2_·xH_2_O) and cerium chloride hydrate (CeCl_3_·xH_2_O), PEG_2k_-NH_2_, 2′,7′-Dichlorodi-hydrofluorescein diacetate (DCFH-DA), methylthiazolyldiphenyl-tetrazolium bromide (MTT), and Calcein/PI Cell Viability/Cytotoxicity Assay Kit were brought from Shanghai Beyotime Biotechnology Co., Ltd. Annexin V-FITC/7-AAD Cell Apoptosis Kit was purchased from Beijing Baiaolaibo Technology Co., Ltd. Phosphate-buffered saline (PBS) was purchased from Hyclone. ICG was purchased from BBI life science Co., Ltd. DAPI were provided by Shanghai Kanglang Biotechnology Co., Ltd. Hydroxyphenyl fluorescein (HPF) was brought from Beijing Baiaolaibo Technology Co., Ltd. Fetal bovine serum (FBS), 0.25% trypsin-EDTA, were provided by Gibco. Anti-CRT and FITC goat anti-rabbit IgG antibody and Anti-HMBG1 and FITC goat anti-rabbit IgG antibody, anti-mouse CD3-APC, CD8-PE, CD4-FITC, CD11c-FITC, CD80-APC and CD86-PE were brought eBioscience. The deionized (DI) water was purified and prepared by Milli-Q water (18 MΩ/cm) for the whole experiment.

**2. Characterizations**

The morphologies of nanomaterials were observed by an H-7650 TEM (Hitachi, Japan) at 100 kV. EDS mapping was conducted from Talos F200s (FEI, USA) operated at 200 kV. Specific surface area and micropore structure of nanoplatform were measured by a BSD 3H-2000PM2 analyzer (Bei Shi De, China). The size distribution and zeta potential of nanomaterials were measured using a NanoBook Omni High Sensitivity Particle Sizing and Zeta Potential Analyzer (Brookhaven, USA). The confocal images were acquired on a TCS SP5 CLSM (Leica, Germany). Cell imaging was carried out by a confocal laser scanning microscope (CLSM) (TCS SP5, Leica, Germany). Cell death and T cells analysis for ICD were carried out by a flow cytometry (CytoFLEX, Beckman Coulter, USA In vivo NIR-II fluorescent images were obtained under a NIR-OPTICS Series III 900/1700 system (808 nm laser irradiation with a 1000 nm long filter pass). Phothemral images were obtained by FOTRIC 225s. MRI was carried out by the magnetic resonance imaging system (9.4T MicroMRI, Bruker, Germany).

**3. Experimental section**

*3.1. In vitro cell culture and cell internalization*

*Cell Culture.* Human osteosarcoma cells (143b) purchased from were obtained from Cell Bank in Chinese Academy of Sciences. Cells were cultured in high-glucose DMEM containing 10% fetal bovine serum, 100 units per mL penicillin, and 100 mg mL^−1^ streptomycin nder a 37°C humidified incubator with 5% CO_2_. Further, the medium was changed every 2 days.

*Cellular Uptake.* To test the cellular internalization efficiency against 143b cells, mCu&Ce@ICG/RGD, mCu@ICG/RGD and ICG were performed in 143b cells. Firstly, 1 × 10^5^ 143b cells were seeded into a 12 well plate with glass coverslips. After 12 h incubation, the adherence cells confluence reached 80%, then cells were treated with mCu&Ce@ICG/RGD, mCu@ICG/RGD and ICG (same ICG concentration: 1 μg/ml). Finally, the cells were fixed and stained nuclei by DAPI for 0.5 h. After 3 times washing with 1 × PBS, subsequently, the tumor cells were imaged by CLSM.

*3.2. Cellular killing effect*

*Cell Viability Assay.* The cell killing effect against 143b cells was assessed by using the standard MTT assay. The 143b cells were cultured and lifted as described above before seeding (1 × 10^4^) into 96-well plates and incubating for 12 h. The medium then was replaced with fresh medium containing various concentrations of mCu&Ce@ICG/RGD, after 12 h incubation, all cells were analyzed by MTT.

*Apoptosis Assay.* For further study the mechanism of cell death, the Annexin V-FITC/7-AAD Cell Apoptosis Kit was employed to assess cell death). 143b cells were seeded into 6-well plate and incubated for 12 h in normal oxygen levels (21%). Subsequently, 1 mL culture medium containing PBS, mCu&Ce@ICG/RGD, mCu&Ce@ICG/RGD+L and mCu&Ce@ICG/RGD+L+H_2_O_2_ were added. The laser irradiation groups were subjected to an 808 nm laser illuviation (5 min, 1.5 W/cm^2^) after 12 h incubation in normoxia condition of nanoplatform. After incubation of another 12 h, cells were digested, collected and stained with Annexin V-FITC and 7-AAD for 10 min, following by analyzing *via* flow cytometry.

*Living/dead Cell Assay.* 143b cells were seeded in 12-well plates, after 12 h incubation, the medium then was replaced with fresh medium containing PBS, mCu&Ce@ICG/RGD, mCu&Ce@ICG/RGD+L and mCu&Ce@ICG/RGD+L+H_2_O_2_ were added for 12 h. In the laser irradiated group, cells were irradiated by 808 nm laser (5 min, 1.5 W/cm^2^). After incubation of another 12 h, the cells were stained with Calcein AM (live cells, green)/PI (dead cells, red) for 10 min and immediately imaged by CLSM.

*3.3. Statistical analysis*

Data obtained in the experiment were verified at least 3 times and expressed as means ± SD. All statistical analyses were performed using a two-sided student’s t-test between different groups. Significance was presented as **p* < 0.05, ***p* < 0.01, and ****p* < 0.001.

**4. Supplementary figures and tables**


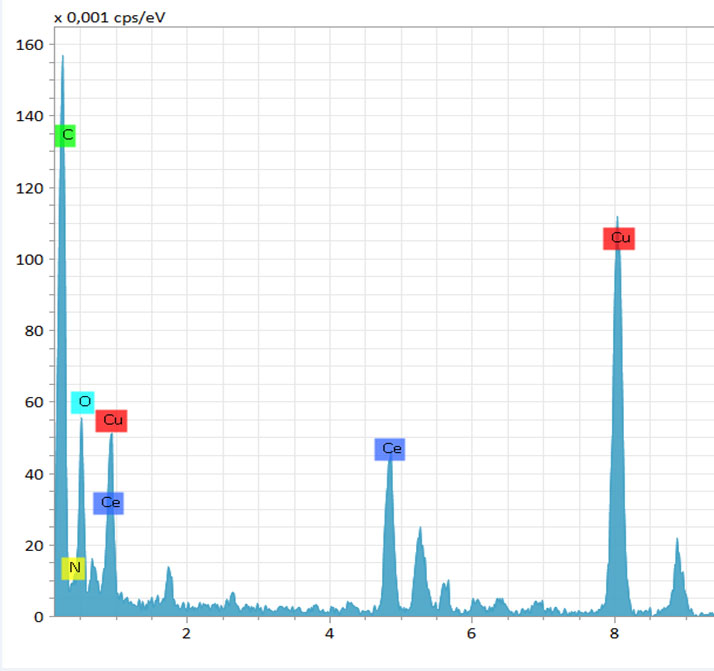


**Figure S1**. Energy dispersive spectra (EDS) image of mCu@Ce.


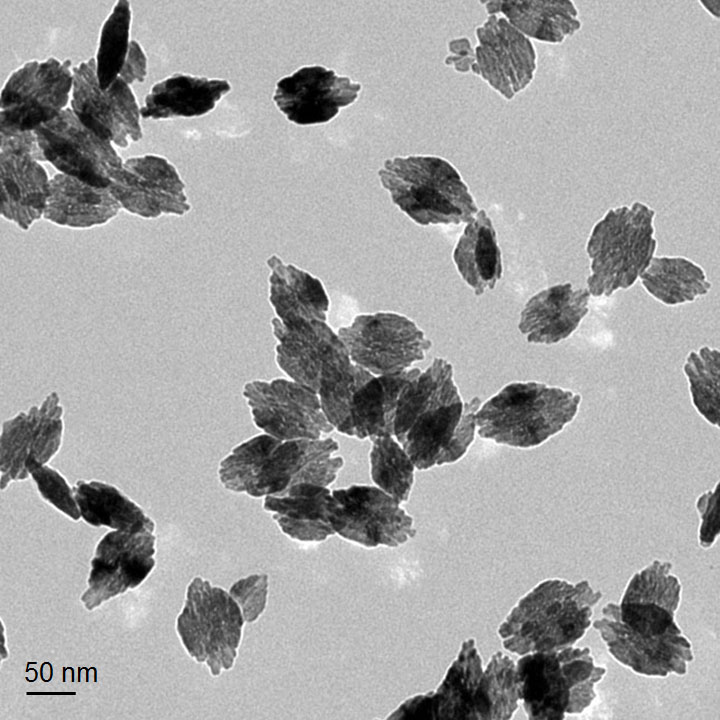


**Figure S2.** TEM image of mCu.


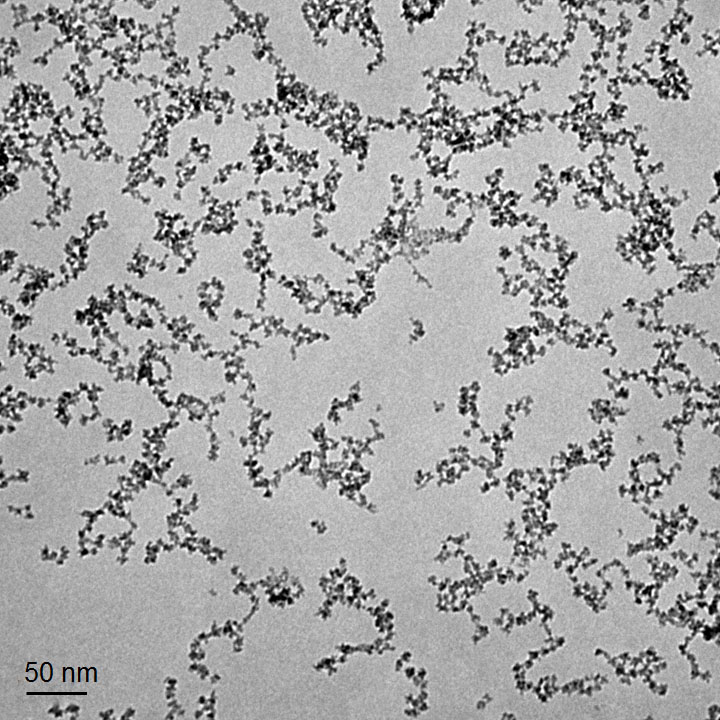


**Figure S3.** TEM image of mCe.


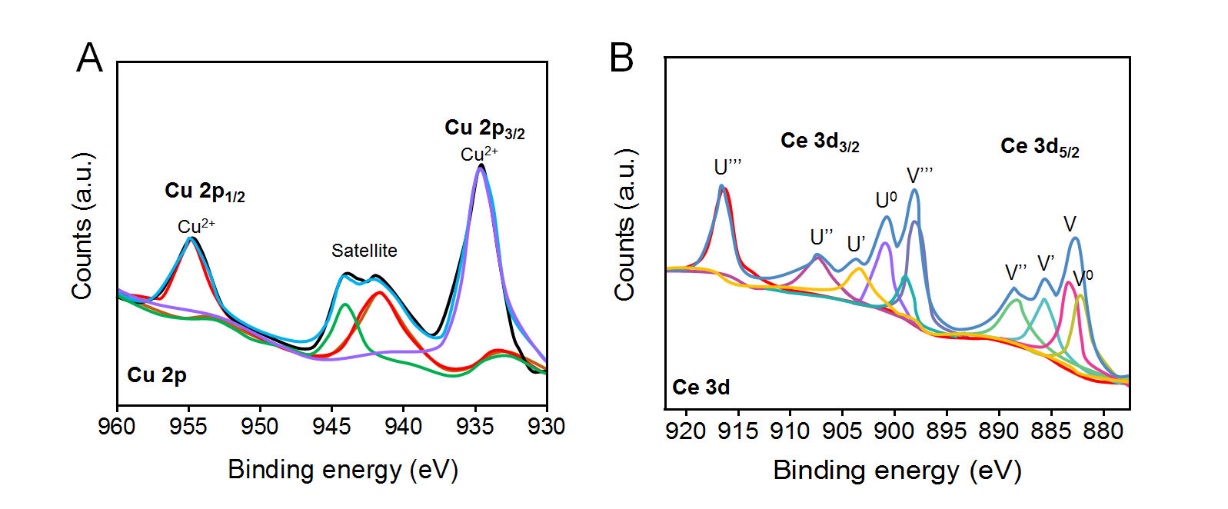


**Figure S4.** Cu 2p (A) and Ce 3d (B) XPS spectra of mCu&C. The peaks marked as U, U’’, U’’’, V, V’’ and V’’’ belonged to Ce^4+^, and other peaks labeled as U^0^, U’, V^0^ and V’ are related to Ce^3+^.


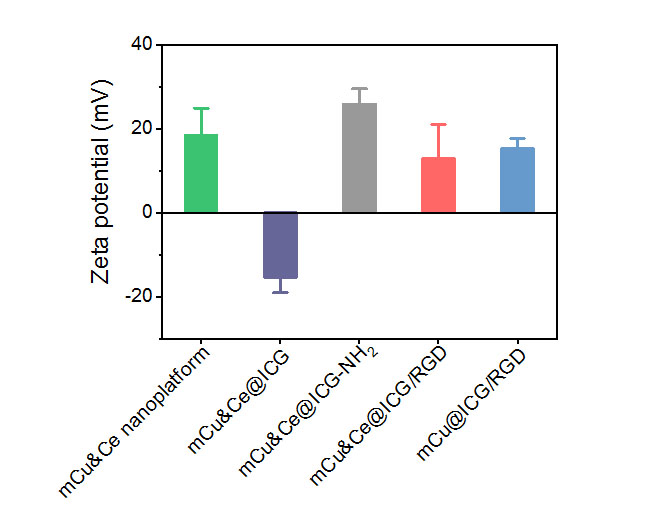


**Figure S5.** Zeta potential data of various nanoformaulations during the synthesis of mCu@Ce@ICG/RGD.


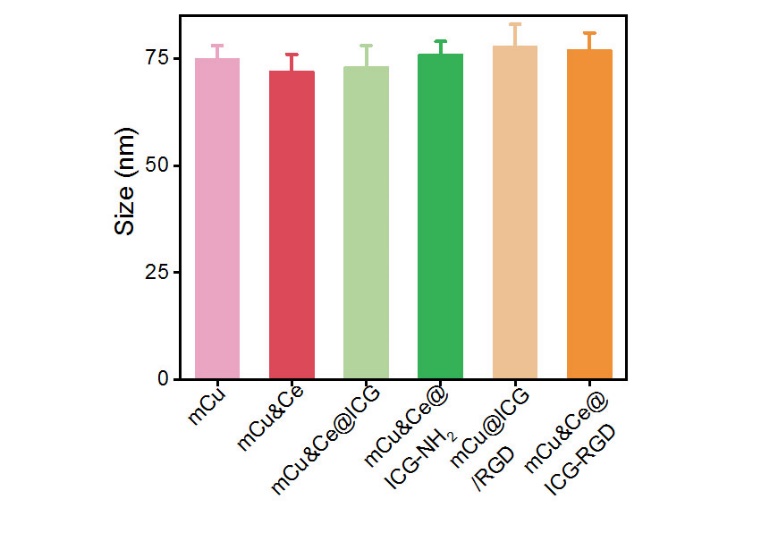


**Figure S6.** Size variations of various Cu based nanoparticles.


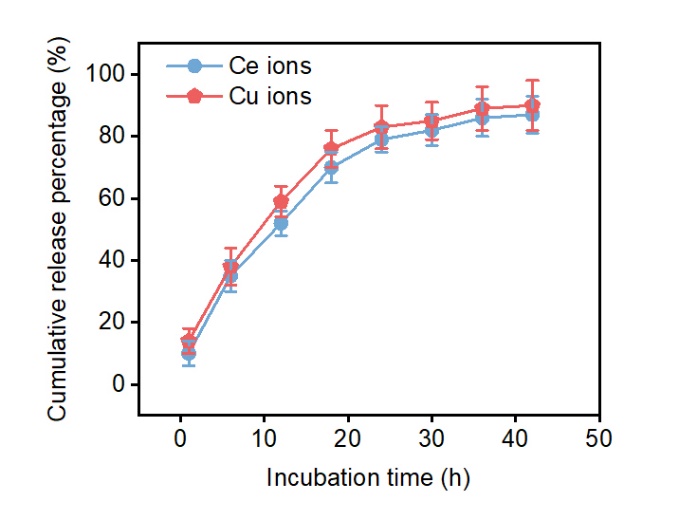


**Figure S7.** Cu and Ce ions released trends of mCu&Ce@ICG/RGD incubated with pH= 6.5 buffers for various hours.


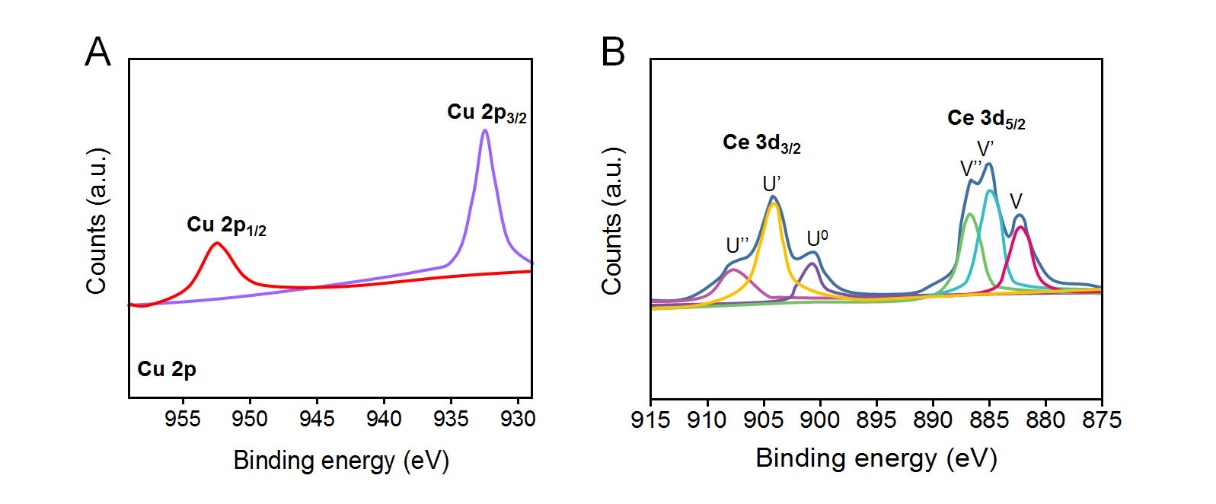


**Figure S8.** Cu 2p (A) and Ce 3d (B) XPS spectra of mCu&Ce@ICG/RGD+H_2_O_2_+L.The peaks marked as U’’, V, and V’’ belonged to Ce^4+^, and other peaks labeled as U’, U^0^ and V’ are related to Ce^3+^.


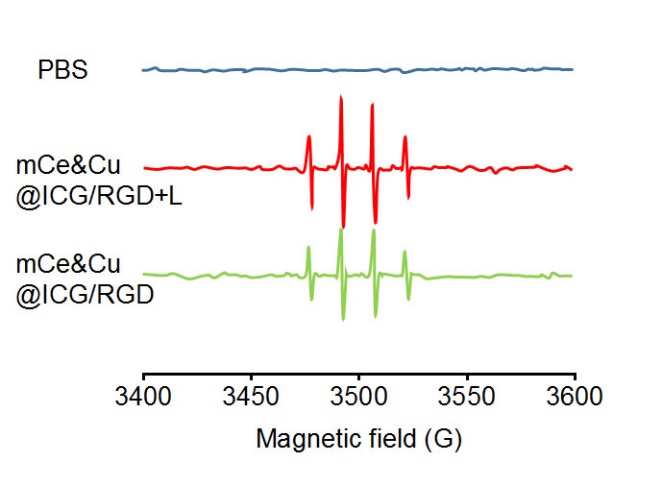


**Figure S9.** EPR spectra of PBS, mCu&Ce@ICG/RGD, and mCu&Ce@ICG/RGD+L under high H_2_O_2_ condition.


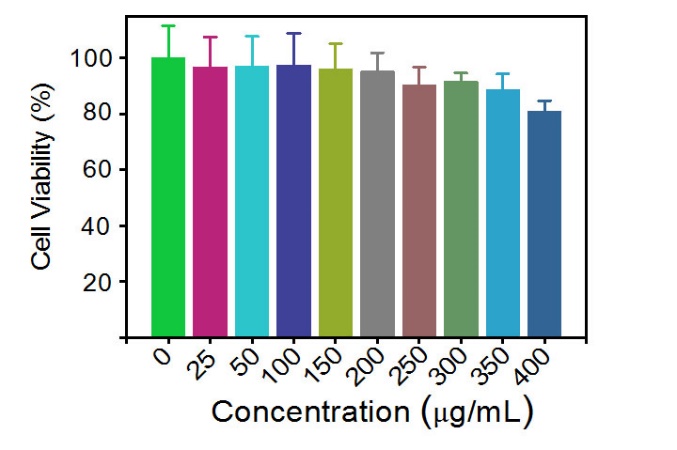


**Figure S10.** Cell viability of 143b cells after co-cultured with mCu@Ce@ICG/RGD under different concentrations.


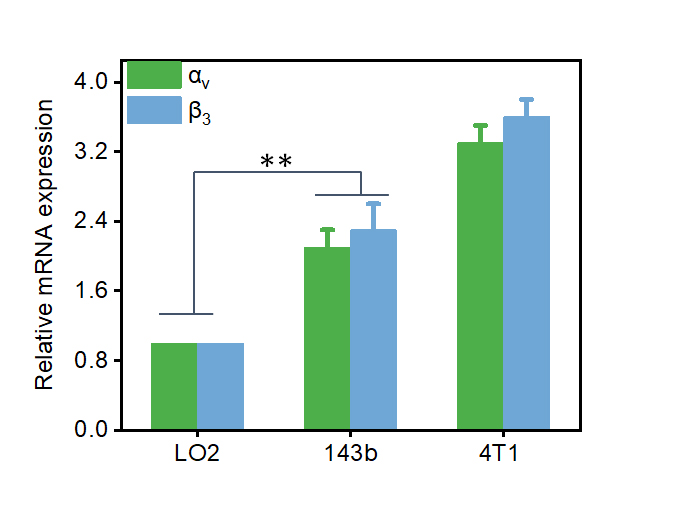


**Figure S11**. Relative α_v_ and β_3_ mRNA expressions in 143b cell, normal liver cells (LO2, negative group) and mammary carcinoma cells (4T1, positive group) are analyzed by real-time quantitative PCR. **p<0.01.


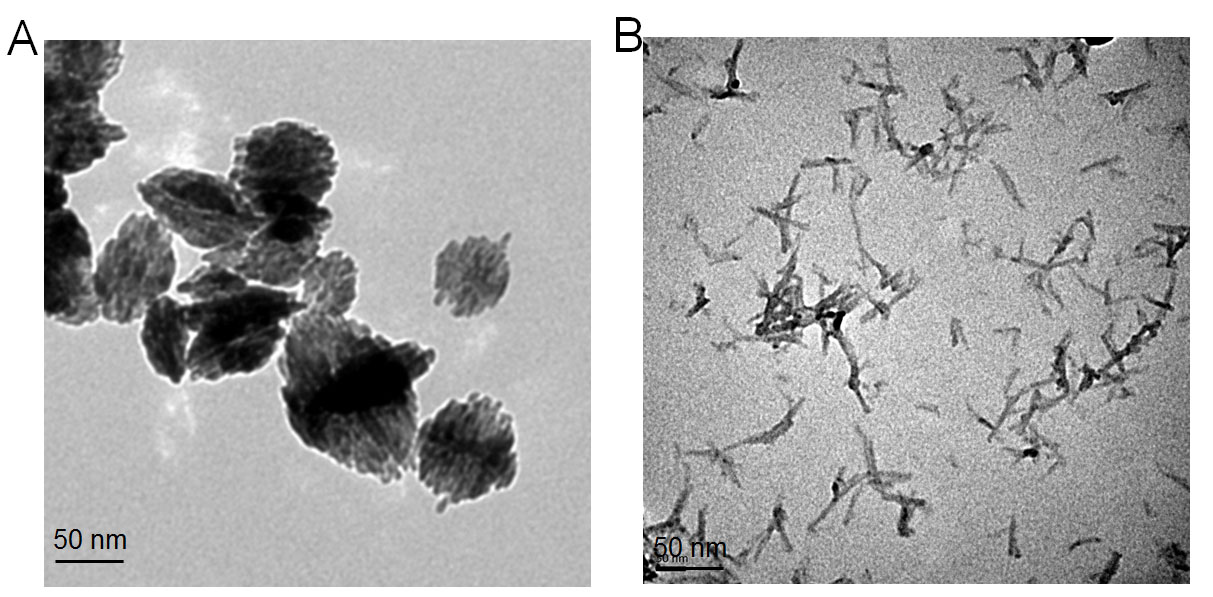


**Figure S12.** TEM images of mCu before (A) and after immersion with pH=6.5 buffer for 36 h.


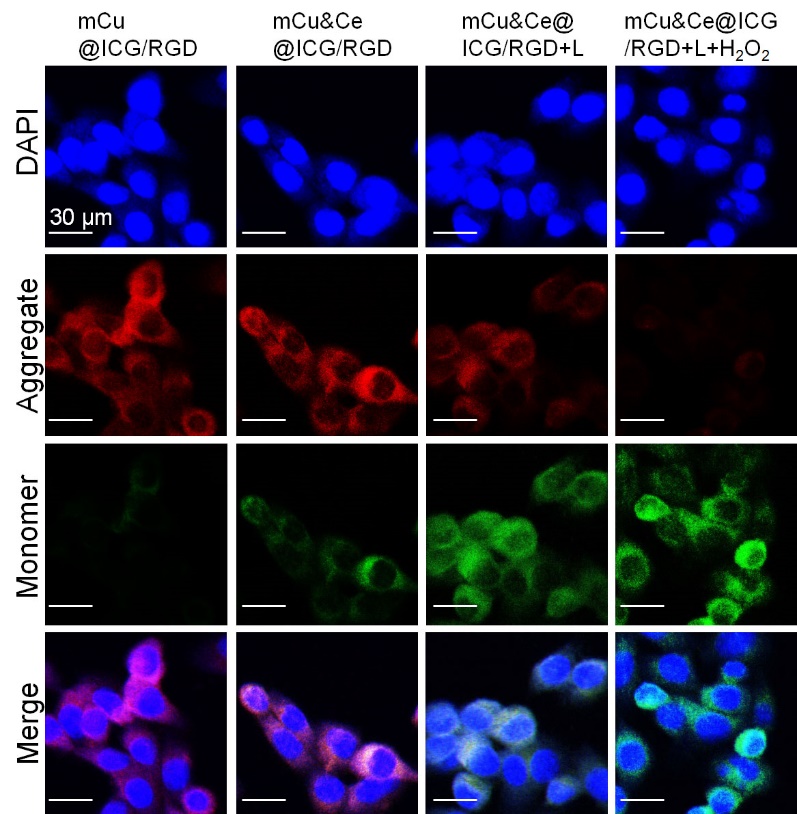


**Figure S13.** CLSM images of mitochondrial membrane potential evauated by JC-1 staining after treated with various nanoformulations.

**
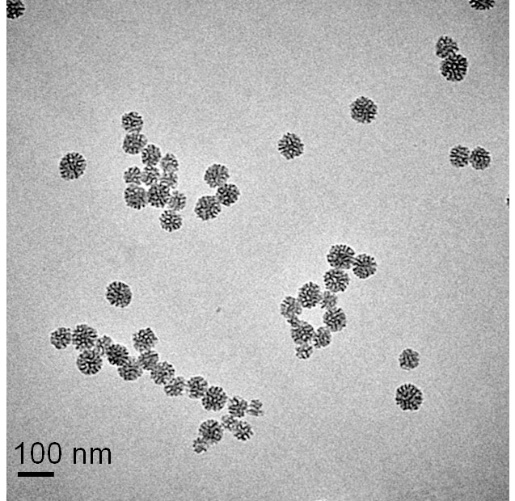
**

**Figure S14.** TEM image of MSN@ICG/RGD.


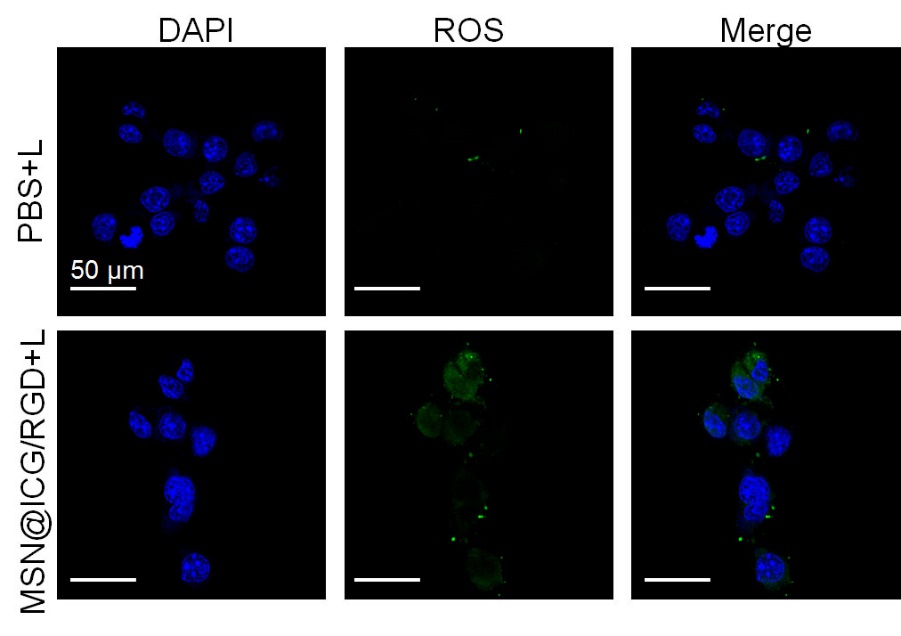


**Figure S15.** CLSM images of cytoplasmic ROS generation in 143b cells after administrated with MSN@ICG/RGD+L.


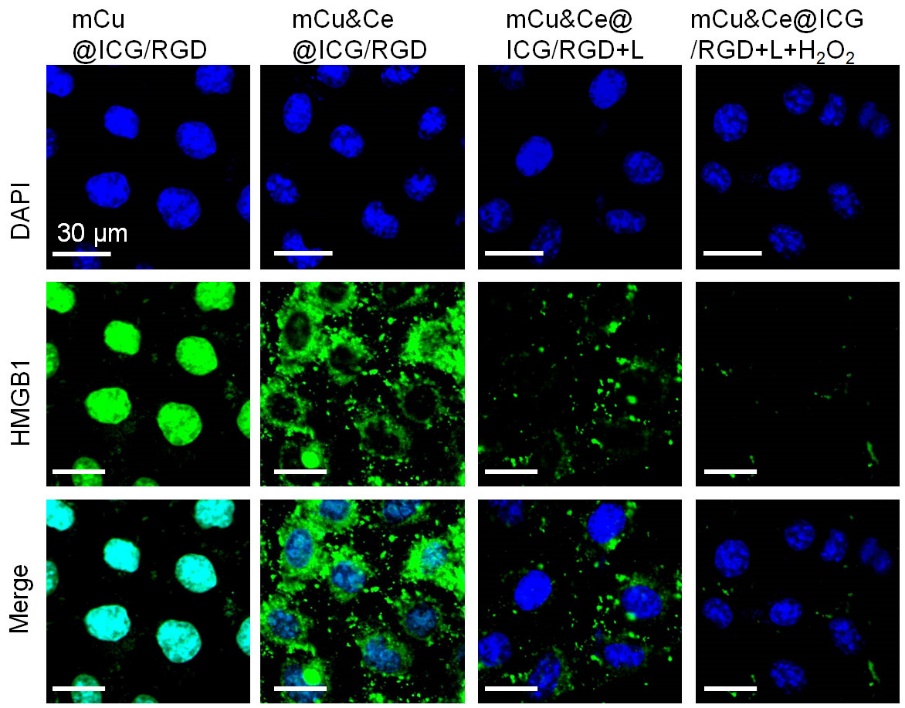


**Figure S16.** CLSM images of cytoplasmic HMGB1 expression in 143b cells after treated with mCu@ICG/RGD, mCu&Ce@ICG/RGD, mCu&Ce@ICG/RGD+L and mCu&Ce@ICG/RGD+L+H_2_O_2_.


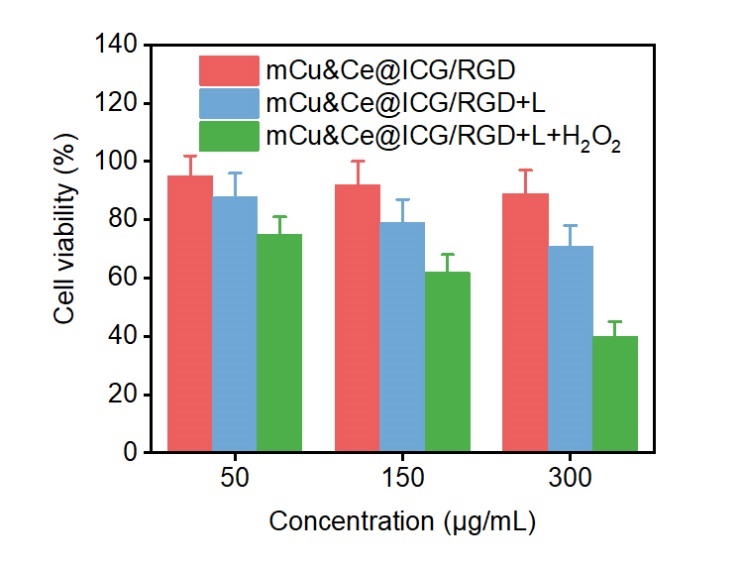


**Figure S17.** Cell viability of 143b cells after treated with various formulations under different concentrations.


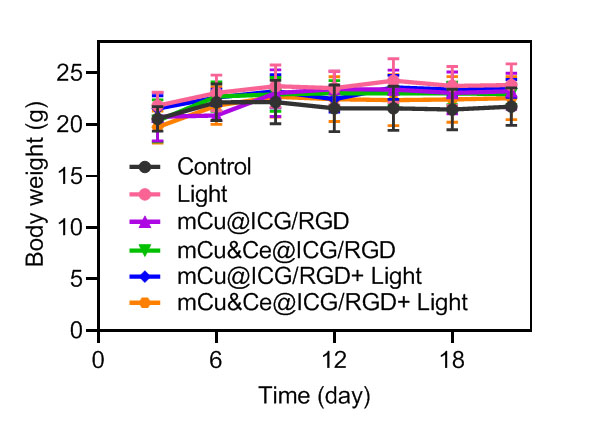


**Figure S18.** Body weight monitoring during the whole treatment of various administrations.


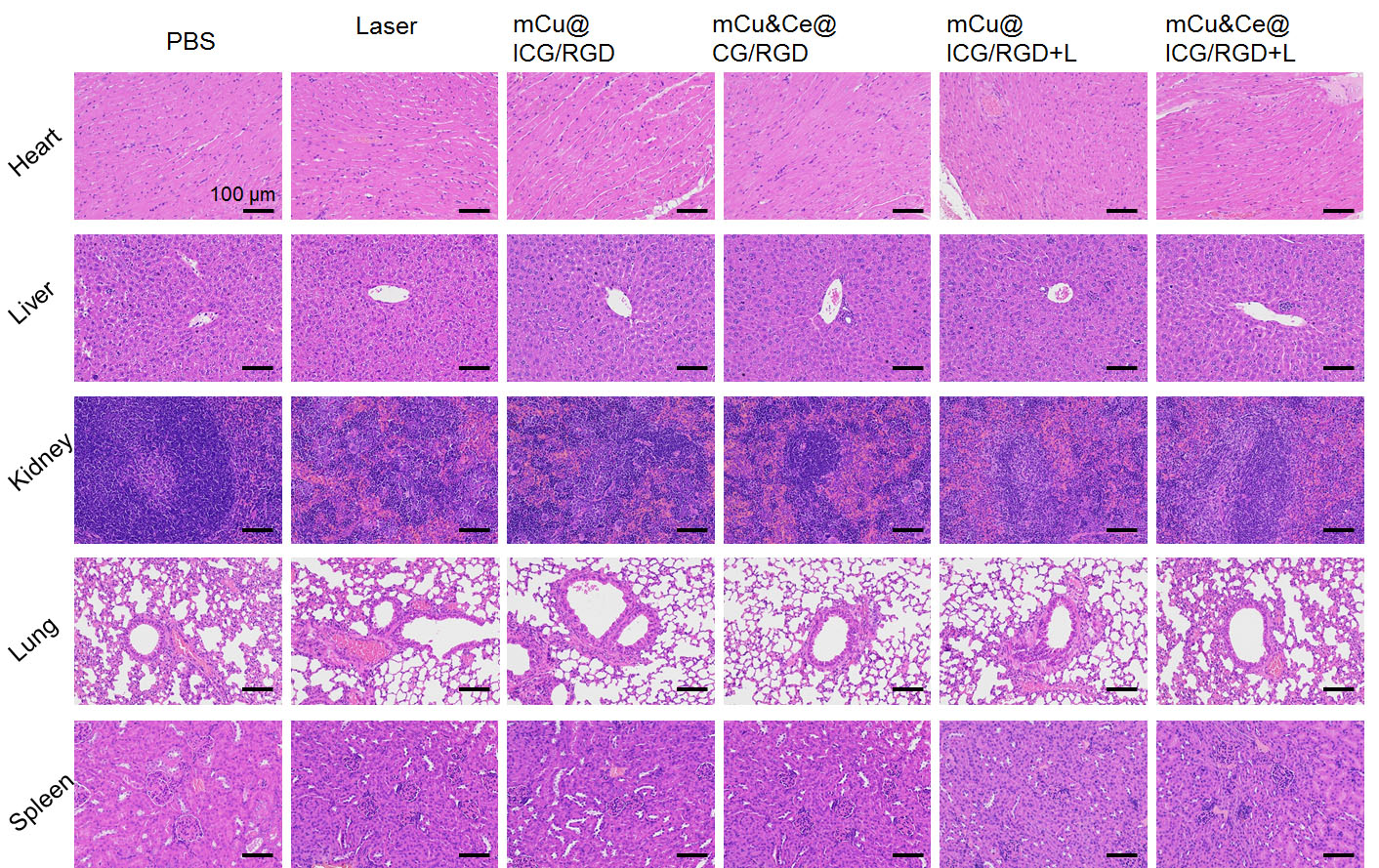


**Figure S19.** H&E stained images of the main organs (heart, liver, kidney, Lung and Spleen) at the final time point (21 days) of various administrations.


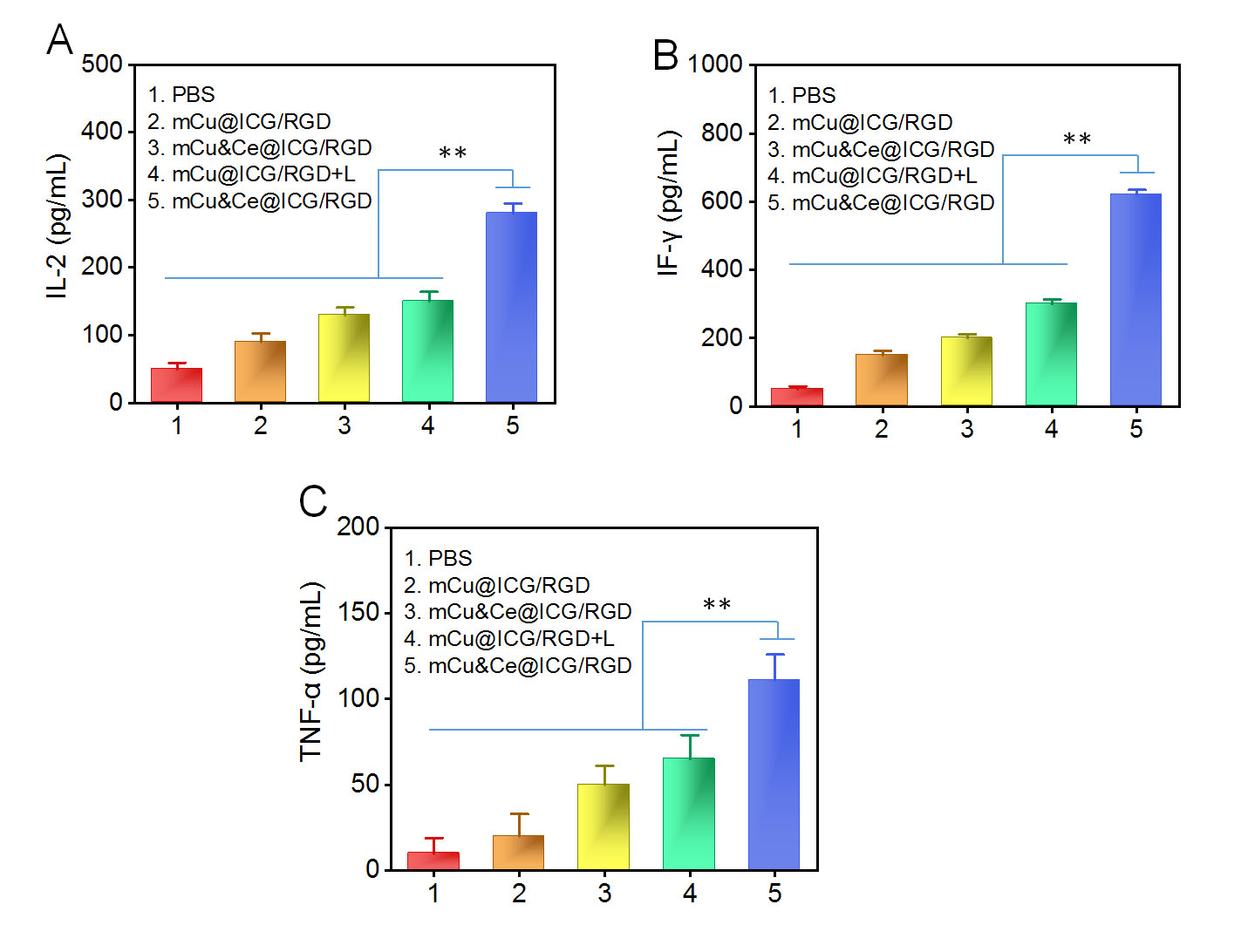


**Figure S20.** Serum levels of inflammatory cytokines IL-2 (A), IFN-γ (B) and TNF-α (C) after different treatments. **P < 0.01.
